# The pleiotropic effects of decanoic acid treatment on mitochondrial function in fibroblasts from patients with complex I deficient Leigh syndrome

**DOI:** 10.1007/s10545-016-9930-4

**Published:** 2016-04-14

**Authors:** Marta Kanabus, Elisa Fassone, Sean David Hughes, Sara Farahi Bilooei, Tricia Rutherford, Maura O’ Donnell, Simon J. R. Heales, Shamima Rahman

**Affiliations:** 1 Genetics and Genomic Medicine, UCL Institute of Child Health, 30 Guilford Street, London, WC1N 1EH UK; 2 Chemical Pathology, Great Ormond Street Hospital for Children NHS Foundation Trust, London, WC1N 3JH UK; 3 Vitaflo International, Liverpool, UK; 4 Neurometabolic Unit, National Hospital for Neurology and Neurosurgery, London, WC1N 3BG UK; 5 Metabolic Department, Great Ormond Street Hospital Foundation Trust, London, WC1N 3JH UK

## Abstract

There is growing interest in the use of the ketogenic diet (KD) to treat inherited metabolic diseases including mitochondrial disorders. However, neither the mechanism whereby the diet may be working, nor if it could benefit all patients with mitochondrial disease, is known. This study focusses on decanoic acid (C10), a component of the medium chain triglyceride KD, and a ligand for the nuclear receptor PPAR-γ known to be involved in mitochondrial biogenesis. The effects of C10 were investigated in primary fibroblasts from a cohort of patients with Leigh syndrome (LS) caused by nuclear-encoded defects of respiratory chain complex I, using mitochondrial respiratory chain enzyme assays, gene expression microarray, qPCR and flow cytometry. Treatment with C10 increased citrate synthase activity, a marker of cellular mitochondrial content, in 50 % of fibroblasts obtained from individuals diagnosed with LS in a PPAR-γ-mediated manner. Gene expression analysis and qPCR studies suggested that treating cells with C10 supports fatty acid metabolism, through increasing *ACADVL* and *CPT1* expression, whilst downregulating genes involved in glucose metabolism (*PDK3*, *PDK4*). *PCK2*, involved in blocking glucose metabolism, was upregulated, as was *CAT*, encoding catalase. Moreover, treatment with C10 also decreased oxidative stress in complex I deficient (rotenone treated) cells. However, since not all cells from subjects with LS appeared to respond to C10, prior cellular testing in vitro could be employed as a means for selecting individuals for subsequent clinical studies involving C10 preparations.

**Electronic supplementary material:**

The online version of this article (doi:10.1007/s10545-016-9930-4) contains supplementary material, which is available to authorized users.

## Introduction

Mitochondrial diseases are the most common group of inherited metabolic disorders, affecting approximately 1:5000 people (Thorburn 2004). They are unusually heterogeneous diseases, both phenotypically and genetically, and can present at virtually any age and with any symptom or combination of symptoms. To date >200 mitochondrial DNA (mtDNA) and nuclear-encoded genes have been linked to mitochondrial disorders (Rahman 2013). Leigh syndrome, also known as subacute necrotising encephalomyelopathy, is the most severe form of mitochondrial disease. Patients usually present in late infancy or early childhood, with progressive neurological abnormalities. Symptoms include respiratory abnormalities, nystagmus, ataxia, dystonia and hypotonia (Fassone and Rahman 2012; Leigh 1951). Diagnosis of Leigh syndrome requires the presence of characteristic clinical features, increased lactate in blood and/or cerebrospinal fluid, and the appearance of characteristic bilateral symmetrical hyperintensities in the basal ganglia and/or brainstem in T2-weighted magnetic resonance image sequences (Fassone and Rahman 2012; Rahman et al 1996). Leigh syndrome is itself genetically heterogeneous, with causative mutations found in genes encoded by the mtDNA, 14 nuclear-encoded subunits of complex I (Fassone and Rahman 2012), and numerous other genes (Lake et al 2015). Despite many advances made in understanding the molecular causes of mitochondrial diseases, treatment options are still very limited and mainly rely on alleviation of symptoms (Kanabus et al 2014).

The ketogenic diet (KD), a high fat, low carbohydrate diet used to treat drug-resistant epilepsy for decades, has been investigated previously as a potential treatment for patients with mitochondrial diseases. In cybrid cell lines containing mtDNA deletions, the KD was shown to induce a heteroplasmy shift in favour of wild-type mtDNA (Santra et al 2004). Studies in animal models also showed that the KD can trigger various beneficial cellular changes. Increased mitochondrial respiration, decrease of reactive oxygen species (ROS), upregulation of expression of uncoupling proteins (Sullivan et al 2004), and increase of glutathione and lipoic acid levels (Jarrett et al 2008) were observed in mice fed with the KD. In the Deletor mouse (a mouse model for mitochondrial diseases, with mutations in the Twinkle helicase which results in multiple mtDNA deletions) a decrease in COX-negative fibres, improved mitochondrial ultrastructure and increased mitochondrial biogenesis were also observed (Ahola-Erkkila et al 2010). The few clinical reports of the use of KD or high fat diet in complex I deficiency noted improvement in seizures (Kang et al 2006; Seo et al 2010; Yoon et al 2014), and transient improvement of oculomotor palsy in one patient with early-onset Leigh-like syndrome due to *NDUFV1* mutations (Laugel et al 2007). However, long-term follow up and treatment of larger cohorts has not been reported, and formal clinical trials are required to provide evidence of efficacy (Rahman 2012).

The KD mechanism of action remains poorly understood, despite its use in clinical settings for decades. Proposed mechanisms include alterations in mitochondrial antioxidant status (Jarrett et al 2008), modulation of neurotransmitter levels (e.g. gamma-aminobutyric acid, GABA) (Yudkoff et al 2004) and changes to cellular energy metabolism accompanied by mitochondrial biogenesis (Bough et al 2006). The medium-chain triglyceride (MCT) diet is a variation of the classic KD. It is as effective in treating seizures as the classic KD, which mostly consists of long-chain fats. In the classic KD approximately 80 % of total caloric intake is fat derived, whereas in the MCT diet this proportion can be reduced to 60 % (Neal et al 2009), and it predominantly contains octanoic acid and decanoic acid, which are also observed in increased levels in the plasma of individuals on this diet (Haidukewych et al 1982).

**Fig. 1:**
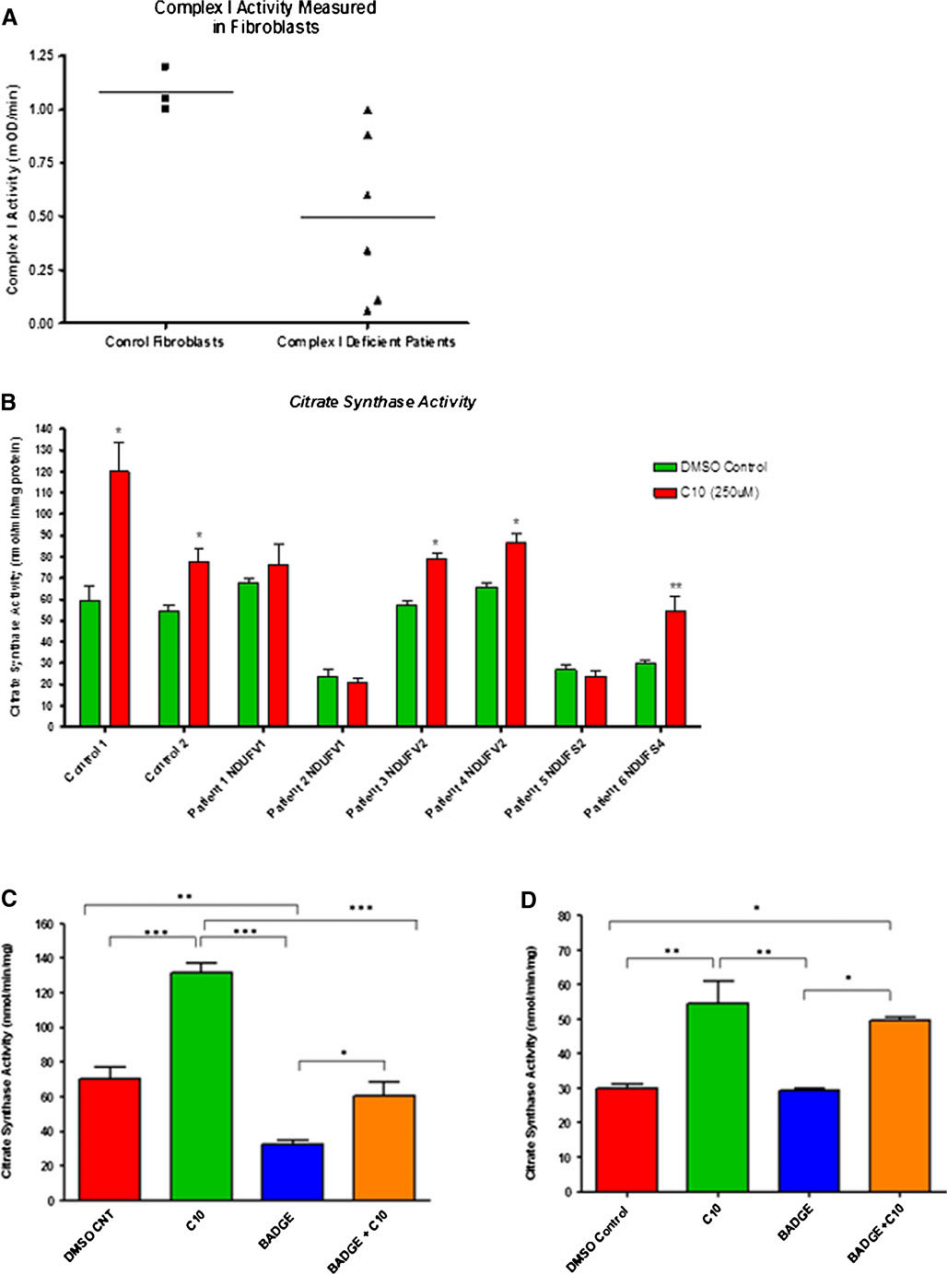
**a** Complex I activity measured in fibroblasts obtained from healthy individuals or complex I deficient patients diagnosed with Leigh syndrome. **b ** Both control fibroblast samples showed a significant increase in CS activity following C10 treatment. Fibroblasts from subjects 1 and 2 (siblings, compound heterozygous *NDUFV1* mutations) showed a varied CS response following C10 exposure. Cells from subject 1 showed a significant increase in CS activity, whereas cells from subject 2 did not. Subject 6 (homozygous mutation in *NDUFS4*) and subjects 3 and 4 cells (sib-pair, with homozygous mutation in *NDUFV2*) all showed a significant increase in CS activity following C10 treatment. Subject 5 cells (with compound heterozygous mutations in *NDUFS2*) did not show a change in CS enzyme activity. *p < 0.05, **p < 0.01. **c** CS enzyme activities measured in control 1 fibroblast cells. Cells were incubated with C10, BADGE or both C10 + BADGE. Incubation with C10 increased CS enzyme activity by 100 % compared to control. Incubation with BADGE significantly decreased CS activity compared to control, whereas co-incubation of BADGE + C10 restored CS activity to control levels; **d** CS enzyme activities measured in subject 6 fibroblast cells. Incubation with C10 increased CS enzyme activity by twofold. Incubation with only BADGE did not have an effect on CS activity; however, incubation with BADGE + C10 resulted in CS activity similar to that obtained with just C10 incubation. *p < 0.05, **p < 0.01, ***p < 0.001

Recently decanoic acid (C10) was reported to be a ligand for the nuclear receptor PPAR-γ which is known to be involved in mitochondrial biogenesis (Malapaka et al 2012). Furthermore C10 has been shown to induce mitochondrial biogenesis and increase complex I and catalase activity in a neuronal cell line (Hughes et al 2014). In light of very few promising treatments on the horizon for primary mitochondrial disorders (Kanabus et al 2014), we sought to determine the effects of C10 treatment on mitochondrial function in primary fibroblasts from a cohort of individuals with nuclear-encoded complex I deficient Leigh syndrome (Table 1 & Fig. 1a).

**Table 1:** Genetic defects of cell lines investigated in this study

Sample	Mutated gene	Biochemistry Complex I activity measured in muscle (normalised to CS activity) (reference range 0.104–0.268) [% mean control]
Patient 1^a^	*NDUFV1*	0.063 [34 %]
Patient 2^ac^	*NDUFV1*	ND
Patient 3^b^	*NDUFV2*	0.067 [36 %]
Patient 4^b^	*NDUFV2*	ND
Patient 5	*NDUFS2*	ND
Patient 6	*NDUFS4*	0.052 [28 %]

^a^patients 1 and 2 are siblings

^b^patients 3 and 4 are siblings

^c^fetal cells; ND not determined (because patients 2 and 4 were siblings of cases who had already been diagnosed, and patient 5 presented before respiratory chain assays were routinely performed in muscle)

## Materials and methods

### Cell culture and treatment

Skin biopsies for fibroblast culture from affected research participants (herein also referred to as subjects) were performed after informed parental consent including consent for research studies. This study received ethical approval from the National Research Ethics Committee London Bloomsbury, UK. Subject 2 cells are fetal cells.

For citrate synthase, complex I and complex IV assays, human skin fibroblast cells (passage 8–15) from controls and subjects were maintained in 182 cm^2^ flasks at 37 °C, 5 % CO_2_ in Dulbecco’s Modified Eagle’s Medium (DMEM), containing 25 mM glucose, 3.97 mM L-Glutamine and supplemented with 10 % heat inactivated fetal bovine serum (FBS, Invitrogen), 50 mg/L uridine and 100 μg/ml penicillin/streptomycin.

For treatment with C10, 100 μl of 50 mM C10 in DMSO (which functioned as the delivery vehicle for C10) was added to 20 ml of supplemented DMEM to a final concentration of 250 μM C10. This concentration was determined as optimal through a dose response curve (data not shown). Untreated fibroblasts were incubated with 100 μl of DMSO alone. All treatments lasted 6 days, with one medium change on day 3. A 6 day incubation with decanoic acid was chosen since it was found to be the optimal time of treatment in time course experiments previously reported by our group (Hughes et al 2014). For some incubations with 250 μM C10, Bisphenol A diglycidyl ether (BADGE, Tocris Bioscience, Bristol, UK), a PPAR-γ antagonist, was added to a final concentration of 25 μM (Hughes et al 2014). Effects of supplementing cells for 6 days with the ketone bodies β-hydroxybutyrate (BHB) or acetoacetate (ACA), both at a final concentration of 5 mM (levels seen in blood of patients on KD) were also investigated.

For L-lactic acid quantification, fibroblasts were grown in six well tissue culture plates and maintained in 2 mL of non-phenol red DMEM medium, containing 25 mM glucose, 3.97 mM L-Glutamine and 25 mM N-2-hydroxyethylpiperazine-N-2-ethane sulphonic acid (HEPES). DMEM was supplemented with 10 % heat inactivated fetal bovine serum (FBS), 100 μg/ml penicillin/streptomycin (10U/μl penicillin G sodium, 10 μg/μl streptomycin sulphate in 0.85 % saline) (Sigma), uridine (final concentration 50 mg/L) (Sigma) and sodium pyruvate (final concentration 1 mM) (Gibco). Cells were treated with C10 or DMSO for 6 days, with one medium change on the third day. On day 6, medium and treatment were changed again, and cells incubated for a further 24 h.

### Assays of citrate synthase, complex I and complex IV activities

Citrate synthase (CS, EC 4.1.3.7) activity was determined according to the method described by Shepherd and Garland (1969), adjusted to be compatible with a 96-well microplate reader. The reaction final volume was reduced to 200 μl. All reaction substrates were proportionally reduced (see Supplemental Methods for details). Sample protein concentration was measured using the Bradford method and adjusted to 1 mg/ml before CS activity determination.

Complex I (EC 1.6.5.3) and complex IV (EC 1.9.3.1) activities were measured using the Complex I Enzyme Activity Microplate Assay Kit and Complex IV Enzyme Activity Microplate Assay Kit respectively (Abcam, Cambridge, UK). Activities of both complexes were measured in whole cell lysates, prepared according to manufacturer’s guidelines.

### L-lactic acid quantification

L-Lactic acid was measured as described by (Gutmann and Wahlefeld 1974), with several adjustments to allow measurements in extracellular fluid from fibroblast cultures using a plate reader system. 200 μl medium from each well was deproteinised by centrifuging with 10 μl of 30 % ZnSO_4_ at 13,000 rpm for 10 min, and the supernatant was transferred into a clean tube and kept on ice. The sample was diluted in water 1:10, and 100 μl of the diluted sample was mixed with 100 μl of the reaction mixture (0.5 M Glycine (Sigma), 1 M Hydrazine-HCl (Sigma), 2 mM EDTA (Sigma), pH 9.3, plus 2 % NAD (Sigma)) in a 96 well plate. Baseline absorbance was measured at 340 nm in a Tecan 200 Pro plate reader; 5 μl of lactate dehydrogenase (LDH, Roche) was added into each well, and absorbance was read again 120 min later. Absorbance values were normalised to the baseline absorbance.

### Mitochondrial volume, membrane potential and ROS

Mitochondrial volume, membrane potential and ROS were measured by a FACS Calibur flow cytometer. Cells were grown and treated for 6 days in 75 cm^2^ flasks. After completion of 6 day C10 treatment, cells were harvested using 0.05 % trypsin-EDTA and counted by FACS Calibur. To-Pro3 (Life Technologies) at a final concentration of 0.05 nM was used to determine cell viability. Staining with either MitoTracker Green FM (final concentration 200 nM), TMRE (final concentration 600 nM), or MitoSox (final concentration 5 μM), was used to measure mitochondrial volume, membrane potential or ROS respectively, per 50,000 cells. Cells were incubated with these stains in a 96-well plate for 15 min at 37 °C, 5 % CO_2_. Cells were washed with PBS (10 % FBS), and resuspended in an equal volume of PBS (10 % FBS) + 0.05nM To-Pro3. Samples were protected from light and immediately transferred onto a flow cytometer fitted with 488 nm wavelength argon and 635 nm wavelength diode lasers. MitoSox and TMRE emission was collected using the 585/42 band pass filter (FL-2) and MitoTracker Green FM using the 530/30 band pass filter (FL-1). To-Pro-3 was measured using the 661/16 band pass (FL-4, red) filter. Data were collected using CellQuest software (BD Biosciences, USA) on a minimum of 10,000 live events, and analysed using the FloJo v.10.0 software (TreeStar, USA).

### Mitochondrial resistance to rotenone induced oxidative stress

Control 1 and subject 6 cells (chosen due to their strongest increase in CS enzyme activity in response to treatment with C10) were grown and treated with 250 μM C10 for 6 days in 75 cm^2^ flasks. On day 5 (24 h before harvesting), the cells were treated with varied concentrations of rotenone, ranging from 0 to 2000 nM in DMSO. Harvested cells were stained with MitoSox at a final concentration of 5 μM and incubated for 15 min at 37 °C, 5 % CO_2_. Cells were washed with PBS (10 % FBS), and resuspended in an equal volume of PBS (10 % FBS). Samples were protected from light and immediately transferred onto the flow cytometer. ROS was measured by a LSR II flow cytometer (BD Biosciences). MitoSox emission was collected using the 575/26 nm (PE-TexasRed) laser. Data were collected using the FACSDiva software (BD Biosciences, USA) based on a minimum of 10,000 events.

### Gene expression analysis using affymetrix GeneChip 1.0 ST microarray

Differential gene expression in control + DMSO compared to C10 treated cells was analysed in control 1 fibroblasts using the GeneChip Gene 1.0 ST Array System (Affymetrix) according to the manufacturer’s guidelines. Fibroblasts were grown in 75 cm^2^ cell culture flasks and supplemented with C10 or DMSO (*n* = 6 C10 group, and *n* = 5 control group) as described above. RNA was extracted using the RNeasy Plus Mini Kit (Qiagen), according to the manufacturer’s instructions. Raw data were normalised according to manufacturer’s protocols using the Expression Console software. Upon normalizing, data were analysed using GeneSpring GX software (Agilent Technologies). Genes which had a >0.3 fold change in expression (i.e. +/− 30 % of control) and a *p* < 0.05 were considered differentially expressed.

### Gene expression analysis using real-time PCR

Fibroblasts from control 1, subject 1 and subject 6 were cultured in 75 cm^2^ cell culture flasks and supplemented with C10 or DMSO (*n* = 3 for each group) as described above. RNA was extracted using the RNeasy Mini Kit (Qiagen) according to the manufacturer’s protocol. GoScript Reverse Transcription System (Promega) was used to make cDNA from an equal amount of RNA in all samples. Relative quantification of ten genes (*CS*, *PPARG*, *PPARGC1A*, *NRF1*, *NRF2*, *ERRA*, *CAT*, *ACADVL*, *HMOX1*, *TXNIP*) was carried out using PowerSYBR Green (LifeTechnologies) according to the manufacturer’s guidelines and normalised to two housekeeping genes, *B2M* and *BACT*. Primer sequences are provided in Table S1. Data analysis was performed using StepOne Software v2.3 (Applied Biosciences).

### Statistical analysis

All results are expressed as mean ± standard error of mean (SEM). The n number refers to the number of independent cell culture preparations, all measured in duplicate or triplicate. Statistical analysis between two groups was performed using the paired Student’s *t*-test, where the same sample was compared (for example treated vs. untreated) or using the unpaired *t*-test, when two different samples were compared (subject vs. control). For multiple comparisons, a one-way ANOVA was used followed by a Tukey post-hoc test. Any ratios were transformed ensuring a normal distribution. Results were considered significant when *p* < 0.05.

## Results

### Increased citrate synthase activity in cells from controls and research subjects following 6 day incubation with decanoic acid

CS enzyme activity is a reliable biomarker of mitochondrial enrichment (Larsen et al 2012). Here we measured CS activity in eight independent primary fibroblast cell cultures exposed to C10 for 6 days (Fig. 1b). We show that the two control fibroblast cultures and six fibroblast cell cultures from six research participants with complex I deficiency (Fig. 1a) respond differently to a 6 day exposure to C10. Control cell cultures both showed a significant increase in CS activity, as did cells from three of the six research subjects following C10 treatment (Fig. 1b). CS was not increased following C10 treatment in fibroblasts from the remaining three subjects (Table S2). Thus cells with different genetic defects appear to respond differently to C10 treatment, even though the affected patients have the same clinical and biochemical disease (complex I deficient Leigh syndrome). CS activity was also increased in fibroblasts from two subjects (subjects 2 and 6) in response to 6 day incubation with BHB, while ACA appeared to have no effect (Fig. S1).

### Respiratory chain complex I and IV activities and L-lactic acid production

Our CS activity data suggested the possibility of mitochondrial content enrichment following C10 treatment. We next sought to determine whether the respiratory chain complex activities had changed after C10 treatment. Complex I enzyme activity increased after C10 exposure in control 2 fibroblasts but not in control 1 or any of the subject cells. Importantly we did not observe any reduction in complex I activity in fibroblasts from either controls or subjects following C10 treatment. Complex IV activity was only reduced in subject 6. Neither ACA nor BHB had a consistent effect on control or subject cell complex I and IV activities. However, complex IV activity decreased in cells from subjects 2, 4 and 6 following both treatments (Fig. S2 and Table S2).

We also wished to determine whether C10 treatment had any effect on L-lactic acid production. We observed no changes in L-lactic acid production in either control or subject cells (Fig. S3).

### Citrate synthase activity following Co-incubation with C10 and the PPAR-γ nuclear receptor antagonist bisphenol A diglycidyl ether (BADGE)

Since C10 is a known PPAR-γ receptor agonist (Malapaka et al 2012), we aimed to determine if the observed increase in CS activity in control and subject fibroblasts was due to activation of the PPAR-γ pathway. In control cells, we found that incubation of fibroblasts with BADGE, a PPAR-γ receptor antagonist, reduced CS activity compared to untreated cells, or those incubated with C10 alone. Furthermore, following co-incubation of C10 and BADGE, CS activities were similar to those in untreated cells (Fig. 1c). In subject 6 fibroblasts, BADGE did not appear to have such a strong effect, and no reduction in CS enzyme activity was observed between untreated cells and those incubated only with BADGE (Fig. 1d). However, in cells treated with BADGE + C10, the CS enzyme levels were as high as those cells treated only with C10 (Fig. 1d). Taken together, these observations suggest that in cells from both controls and affected research participants the increase in CS activity following C10 treatment is mediated via the PPAR-γ pathway.

### Mitochondrial volume, reactive oxygen species and membrane potential following C10 treatment

**Fig. 2:**
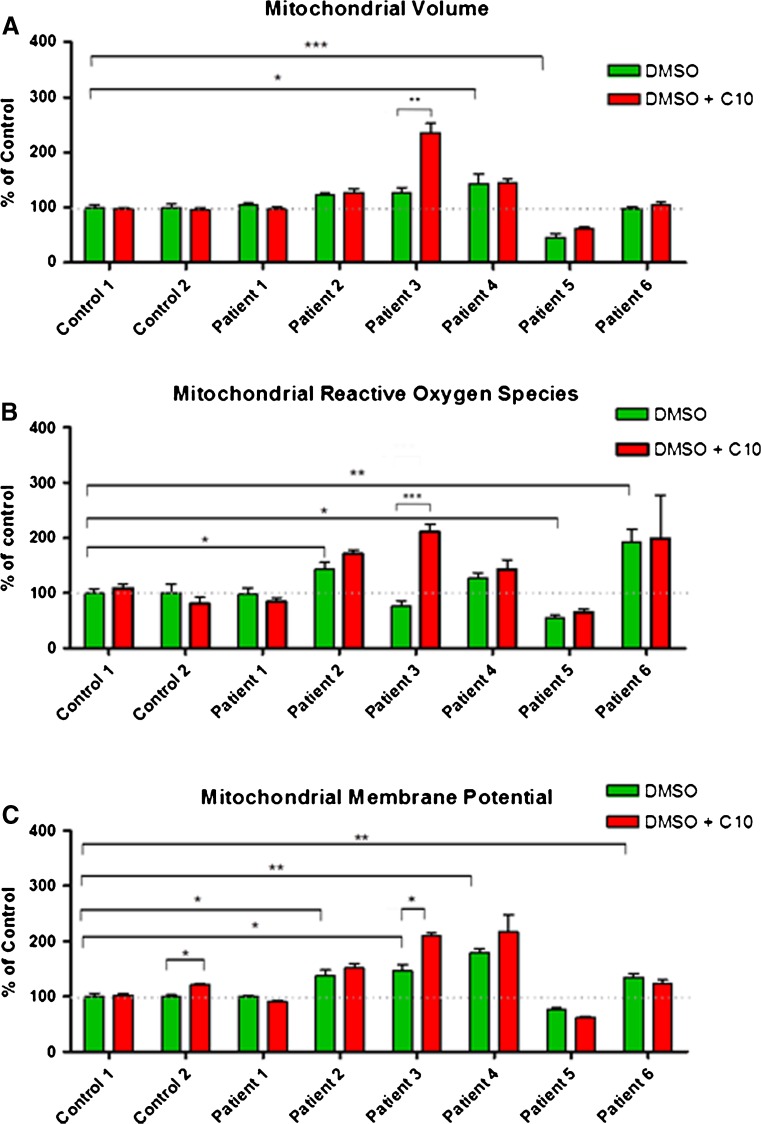
**a** Mitochondrial volume: compared to controls only subject 5 cells showed a reduction in baseline mitochondrial volume. Subject 4 fibroblasts had increased baseline mitochondrial volume compared to control. In response to treatment with C10, only fibroblasts from subject 3 showed a significant increase. **b** Mitochondrial ROS: subjects 2 and 6 fibroblasts showed higher baseline ROS levels when compared to controls. Subject 5 cells had significantly lower baseline ROS when compared to controls. In response to treatment with C10, only subject 3 fibroblasts showed an increase in ROS. **c** Membrane potential: when compared to control cells, subjects 2, 3 and 6 fibroblasts all had higher baseline membrane potential. In response to treatment with C10, control 2 and subject 3 cells showed an increase in membrane potential. Subject 4 cells also appeared to have increased membrane potential when treated with C10; however, this result was not statistically significant. *p < 0.05, **p < 0.01, ***p < 0.001

We found that cells from most research subjects had a similar mitochondrial volume when compared to controls (except subject 4 cells which had a higher mitochondrial volume, and subject 5, with a lower volume) (Fig. 2a). Incubation of fibroblasts with C10 led to a significantly increased mitochondrial volume only in cells from subject 3 (Fig. 2a). Cells from three subjects showed an increase in baseline ROS levels (subjects 2, 4 and 6), compared to ROS levels in healthy control cells (Fig. 2b). Following C10 treatment we found that ROS levels increased significantly in cells from only one subject — subject 3 (Fig. 2b), which also showed baseline increase in mitochondrial membrane potential (Fig. 2c), but because the overall mitochondrial volume in these cells was also increased the ratio of ROS and membrane potential to mitochondrial volume remained unaltered. Additionally an increase in membrane potential was also noted in cells from control 2 (Table S2).

### ROS production in rotenone treated cells and the effects of C10

**Fig. 3:**
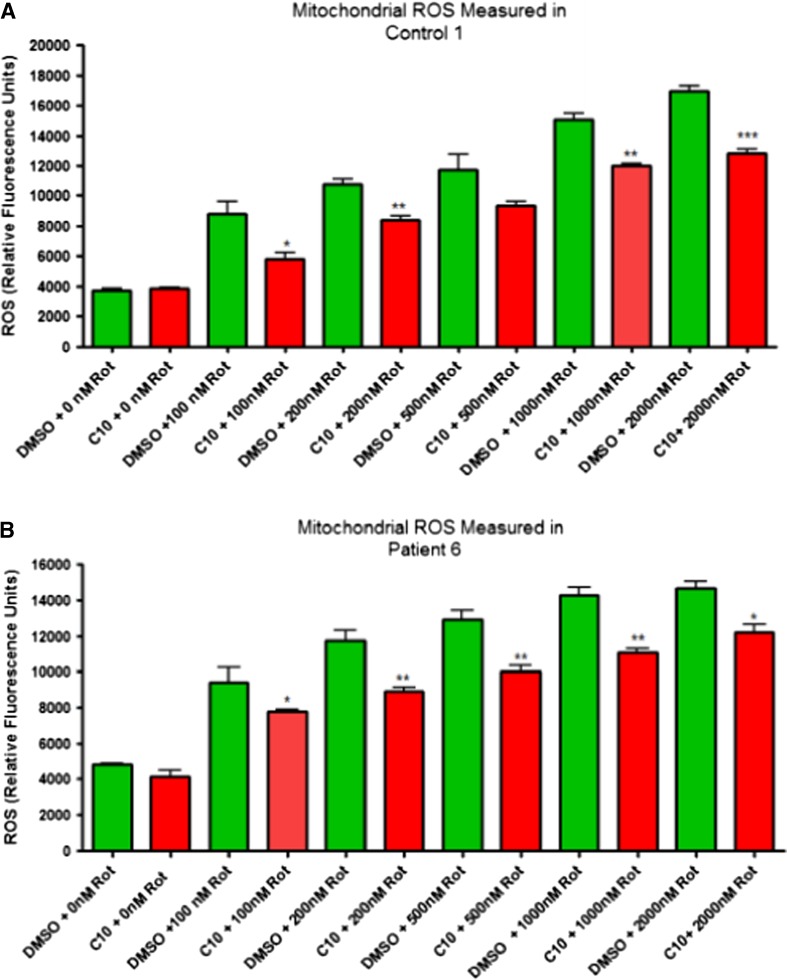
Effects of C10 on mitochondrial oxidative stress. A 6 day treatment with C10 reduced the oxidative stress of control and subject 6 fibroblasts exposed to various concentrations of rotenone for 24 h. C10 did not affect ROS levels in cells not exposed to rotenone. (*n* = 3; *p < 0.05, **p < 0.01, ***p < 0.001)

Based on our previously published finding that C10 increases catalase activity (Hughes et al 2014), and our qPCR data which showed that C10 increased the expression of *CAT*, we aimed to determine whether C10 is able to increase cellular resistance to oxidative stress. This experiment was carried out in control cells, as well as cells from subject 6, as these were the cells which showed the highest increase in CS activity in response to C10. We found that treatment of both the control and subject cells with C10 reduced the amount of ROS triggered by the exposure of the cells to rotenone, but C10 did not decrease ROS levels in cells not exposed to rotenone (Fig. 3).

### Gene expression analysis

In order to better understand the mechanism of action by which C10 is able to increase CS enzyme activity and to gain a more global view of the effects of C10 on fibroblasts, we performed a genome-wide gene expression microarray study. Overall 521 genes had upregulated expression levels and 200 genes were downregulated. None of the common mitochondrial biogenesis genes (such as *PPARG*, *PPARGC1A*, *NRF1*, *NRF2*, *ESRRA*) showed changes in expression levels at the end of a 6 day incubation with C10. The entire list of genes observed to have altered expression can be found in Supplementary Table S3. Genes which where differentially expressed in the two groups, and based on the MitoCarta prediction are expected to encode mitochondrially targeted proteins, are listed in Table 2.

**Table 2:** Mitochondrial genes with differential gene expression levels upon treatment with C10

Genes with upregulated expression levels following C10 treatment	
Gene	Function
*PDK4*	pyruvate dehydrogenase kinase, isozyme 4
*PDK3*	pyruvate dehydrogenase kinase, isozyme 3
*GLYATL2*	glycine-N-acyltransferase-like 2
*ATP5O*	ATP synthase, H+ transporting, mitochondrial F1 complex, O subunit
*CPT1A*	carnitine palmitoyltransferase 1A (liver)
*ACADVL*	acyl-CoA dehydrogenase, very long chain
Genes with downregulated expression levels following C10 treatment	
Gene	Function
*SLC25A23*	solute carrier family 25 (mitochondrial carrier; phosphate carrier), member 23
*PCK2*	phosphoenolpyruvate carboxykinase 2
*MTHFD2*	methylenetetrahydrofolate dehydrogenase (NADP+ dependent) 2 and methenyltetrahydrofolate cyclohydrolase
*DHRS3*	dehydrogenase/reductase (SDR family) member 3
*NDUFC1*	NADH dehydrogenase (ubiquinone) 1, subcomplex unknown, 1,
*ALDH1L2*	aldehyde dehydrogenase 1 family, member L2
*ADHFE1*	alcohol dehydrogenase, iron containing, 1

**Fig. 4:**
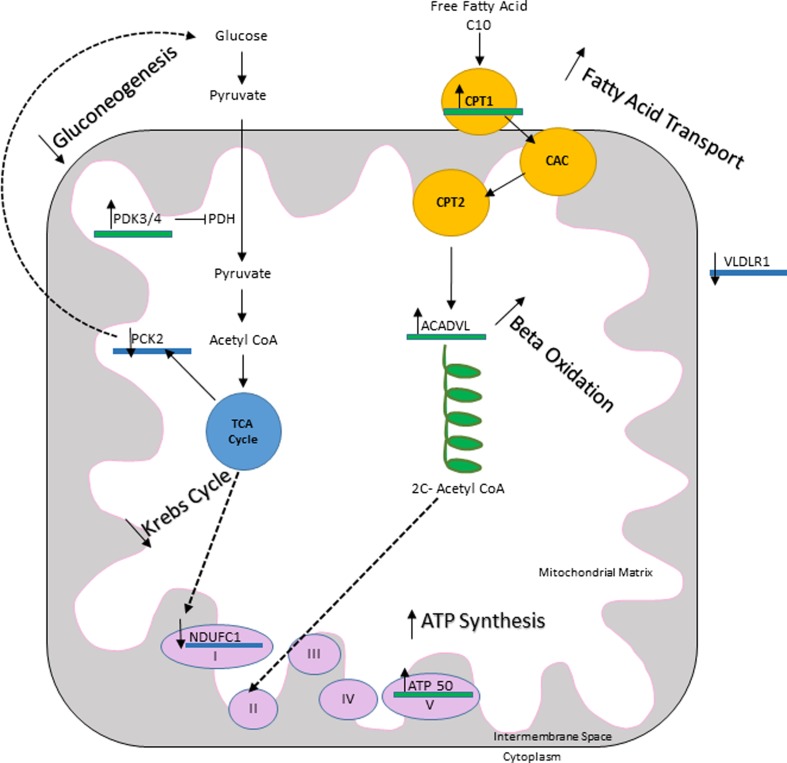
Effects of C10 on gene expression. A 6 day treatment with C10 reduced the expression of genes involved in glucose metabolism, such as *PCK2*, which encodes phosphoenolpyruvate carboxykinase, an enzyme important in the gluconeogenesis pathway. The complex I supernumerary subunit *NDUFC1* (of unknown function), was also reduced. Glucose is metabolised to acetyl-CoA, which enters into the tricarboxylic cycle. Electrons derived from the Krebs cycle enter oxidative phosphorylation through complex I, as opposed to those derived through β-oxidation, which bypass complex I and enter oxidative phosphorylation through complex III. Additionally, *PDK3* and *PDK4* were upregulated. These genes encode the enzyme pyruvate dehydrogenase kinase, which inhibits the enzyme pyruvate dehydrogenase. Genes involved in fatty acid metabolism upregulated after 6 day C10 treatment include *CPT1A* (which encodes a protein responsible for fatty acid transport into mitochondria) and *ACADVL*, which encodes the enzyme VLCAD required for the metabolism of fatty acids with a chain length of 14–20 carbons. *ATP50* (which encodes a subunit of ATP synthase) also showed increased expression in response to treatment with C10

In addition to analysing gene expression through a microarray study, relative quantification of genes known to be involved in mitochondrial biogenesis (*PPARG*, *PPARGC1A*, *NRF1*, *NRF2*, *ESRRA*) was also performed using qPCR. Furthermore, three other genes were also analysed through qPCR in order to validate the microarray results (*ACADVL* and *HMOX1*, which were upregulated in the microarray and *TXNIP*, which was downregulated). We found that relative gene expression levels of these three genes measured in control fibroblasts (the same cells which were used in the microarray study), were in alignment with the microarray data (Fig. S4). Finally, using qPCR we also examined the expression of *CAT*, encoding catalase, and found it to be increased in control cells. This was not observed in the microarray (Fig. 4).

Interestingly, the gene expression levels varied between cells from subjects 1 and 6. Subject 1 cells showed a significant increase in relative expression levels of *HMOX1*, while no change was observed in expression of *TXNIP*. In contrast, cells from subject 6 showed no change in *HMOX1* expression levels, while *TXNIP* was significantly decreased upon a 6 day treatment with C10. *ACADVL* and *CAT* expression was increased in cells from both subjects 1 and 6 after treatment with C10 (Fig. S4).

## Discussion and conclusions

Mitochondrial diseases, despite being the most common group of inherited metabolic disease, are in the majority of cases untreatable and often fatal diseases. Huge scientific efforts are currently being directed towards developing new treatment approaches. However, due to the large genetic and phenotypic heterogeneity of these disorders, validation of new treatments through proper clinical trials is proving to be extremely challenging (Pfeffer et al 2012; Kanabus et al 2014). Several treatment approaches under consideration aim to increase mitochondrial biogenesis, for example Bezafibrate (Yatsuga and Suomalainen 2012) and 5-amino-1-β-D-ribofuranosyl-imidazole-4-carboxamide (AICAR) (Golubitzky et al 2011). Recently our group proposed another alternative, C10, as a potential activator of mitochondrial biogenesis (Hughes et al 2014). It is known that C10, a medium chain fatty acid, is a PPAR-γ ligand (Malapaka et al 2012). Our previous work demonstrated that in a neuronal cell model (SHSY-5Y cells), C10 increased the activities of CS, complex I and catalase (Hughes et al 2014). In view of these findings, in this study we investigated whether C10 could have similar beneficial effects in fibroblast cells obtained from subjects with genetically confirmed primary mitochondrial diseases.

The enzyme CS is widely used as a biomarker for mitochondrial enrichment. It has been shown that CS activity has a strong relationship with mitochondrial fractional area (Larsen et al 2012). We observed an increase in CS activity in the majority of fibroblasts upon incubation with 250 μM C10 for 6 days. Since CS enzyme is a marker for mitochondrial enrichment, we examined overall mitochondrial volume using MitoTracker Green FM staining. We found no significant changes in all but one cell line — subject 3. This suggests that the increase in CS activity may not necessarily be caused by an increase in cellular mitochondrial volume. Furthermore, microarray and qPCR results also did not show an increase in expression levels of *CS*, but a transient change in expression levels may have occurred prior to day 6.

C10 has previously been shown to be a partial activator of the PPAR-γ receptor (Malapaka et al 2012). More potent PPAR-γ activators such as members of the thiazolidinedione drug family (including rosiglitazone) may trigger serious side effects including adipogenesis, stroke and myocardial infarction (Spiegelman 1998; Graham et al. 2010). C10 is a component of the MCT diet, which is not associated with such adverse effects. Additionally, it has been shown that C10 does not increase glucose sensitivity, and improves the overall lipid profile without having an effect on weight in diabetic mice (Malapaka et al 2012). In order to confirm that the increase in CS enzyme activity observed in cells supplemented with C10 is a downstream effect of PPAR-γ activation, we also measured CS activity in the presence of BADGE, a PPAR-γ receptor antagonist (Fig. 1c and d). We found that incubating control fibroblasts in the presence of BADGE reduced CS activity levels significantly, while incubating these cells with BADGE + C10 restored CS activity levels to control values. These data confirm that the observed increase in CS activity occurs as a result of a PPAR-γ mediated pathway, at least in control cells. However, the observation of an attenuated response to BADGE in one subject raises the possibility of altered PPAR-γ mediated signalling in some cases of complex I deficiency.

It has been previously suggested that an increased mitochondrial content may be associated with reduced respiratory chain enzyme activities (Bernier et al 2002). Since we were investigating the effects of C10 on cells obtained from complex I deficient individuals, we measured complex I to ensure that C10 does not have a detrimental effect on these cells. Reassuringly, our results showed that C10 did not exacerbate the complex I deficiency. Moreover, in control 2 cells, complex I activity was increased. Our previous study performed on SHSY-5Y cells also found an increase in complex I activity after treatment with C10 (Hughes et al 2014). Complex I is reported to be rate limiting for ATP formation in neuronal cells but this may not be the case for other cell types such as fibroblasts (Davey et al. 1998). Furthermore, the glycolytic nature of fibroblasts plus the genotype/phenotype of the affected subjects’ cells studied may be contributing factors to the differences seen in this study when compared to those previously performed on a relatively homogeneous cell line.

C10 has a similar chemical structure to the anticonvulsant valproic acid, which has also been linked to increased mitochondrial biogenesis (Hayasaka et al. 1986), and which has been shown to reduce mitochondrial complex IV activity (Ponchaut et al. 1995). It was therefore important also to measure complex IV activity following treatment of the control and subject fibroblasts with C10. We observed a decrease in complex IV activity only in one out of eight fibroblast cultures, suggesting that C10 does not share valproate’s toxic effects on mitochondrial respiratory chain function.

We further evaluated the effects of C10 on ROS levels and mitochondrial membrane potential. Increased ROS levels are thought to be one of the main pathogenic mechanisms underlying mitochondrial disorders, especially in patients with complex I deficiencies, since complex I is a major site of mitochondrial ROS production. Any compound which increases ROS levels could potentially cause further damage to the respiratory chain (Heales et al. 1996; Marí et al. 2009). We found that in all subject cells, except for subject 3, mitochondrial ROS levels were unchanged following C10 treatment. Subject 3 cells also showed an increase in overall mitochondrial volume, which might explain the observed increase in ROS. Indeed, ROS levels per unit of mitochondrial volume were unchanged. Additionally, our qPCR experiments revealed increased *CAT* expression levels in all three cell lines studied, including the one which did not show an increase in CS activity (i.e. cells from subject 1). These data are consistent with our previous observation that C10 treatment increased catalase enzyme activity in SHSY-5Y cells (Hughes et al 2014) and led us to suspect that C10 may increase cellular resistance to oxidative stress. This hypothesis was confirmed by the fact that cells treated with C10 and exposed to rotenone had lower ROS levels than cells not treated with C10 but exposed to the same concentration of rotenone. However, this observation may also indicate an improvement of mitochondrial function as we have previously reported that ROS production is more sensitive than ATP as an indicator of changes in mitochondrial function (Jacobson et al 2005). We also examined mitochondrial membrane potential pre- and post-C10 treatment. Increased mitochondrial membrane potential would be suggestive of better proton pumping functions of the respiratory chain complexes and increased ATP production. It is important to note, however, that increased mitochondrial membrane potential may result in increased ROS levels, and vice versa, high ROS levels can lead to the depolarization of the mitochondrial membrane potential (Korshunov et al. 1997). Furthermore, under certain conditions mitochondrial membrane potential may be maintained at the expense of ATP production, by reversal of the activity of the ATP synthase (Duberley et al. 2013). Similarly to the ROS results, we observed no changes in mitochondrial membrane potential in cells from both controls and all studied subjects, except fibroblasts from subject 3, in which we also observed increased mitochondrial volume, as discussed above.

Our microarray data also suggest that supplementation of cell culture medium with C10 in the presence of high glucose concentration upregulates genes involved in fatty acid metabolism, whilst downregulating expression of genes involved in glucose metabolism (Table 2, Fig. 4). Upregulation of genes such as *LDLR*, *CPT1A* and *ACADVL* suggests that lipid metabolism pathways are overall upregulated without discriminating the length of the fatty acid chain. This may find its applications in a wider range of diseases, such as hyperlipidaemia or obesity. Additionally, we also observed an increase in expression of the *ATP5O* subunit of ATP synthase, alongside a decrease in expression of *NDUFC1*, encoding a supernumerary subunit of complex I, the precise function of which remains unknown. This may reflect the fact that fatty acid metabolism bypasses complex I in the respiratory chain. The genes involved in glucose metabolism which were downregulated include *PCK2* which encodes the enzyme phosphoenolpyruvate carboxykinase, a key enzyme of gluconeogenesis. Additionally, we also observed an increase in expression levels of *PDK4* and *PDK3*, which encode the pyruvate dehydrogenase kinase, an enzyme which inhibits pyruvate dehydrogenase — an essential link between glycolysis and the TCA cycle (Fig. 4). Finally, the observation that expression of genes associated with gluconeogenesis was decreased, whilst that of genes associated with fatty acid oxidation was increased, suggests the possibility that stress signalling may be at play, possibly mediated by AMPK (Hardie et al 2015). This would be an interesting avenue for further investigations.

We conclude that supplementation of fibroblast cells with C10 has pleiotropic effects on mitochondrial function through targeting the PPAR-γ receptor, and thus potentially increasing mitochondrial biogenesis as well as increasing cellular resistance to oxidative stress. Based on these preliminary findings, we suggest that this therapy should now be further investigated in mouse models of mitochondrial disease. Our results also highlight the importance of genetically and phenotypically matched cohorts when studying treatments for mitochondrial diseases, and suggest that not every mitochondrial patient will benefit from the same type of treatment. Furthermore, the assay methods described here could be utilised to identify suitable patients for further investigation of the efficacy of C10 therapy in a clinical trial, in a ‘personalised medicine’ approach.

### Abbreviations

*AICAR* 5-Amino-1-β-D-ribofuranosyl-imidazole-4-carboxamide, *ACA* Acetoacetate, *BADGE* Bisphenol A diglycidyl ether, *BHB* β-Hydroxybutyrate, *C10* Decanoic acid, *GABA* Gamma-aminobutyric acid, *KD* Ketogenic diet, *LDH* Lactate dehydrogenase, *MCT* Medium chain triglyceride, *mtDNA* Mitochondrial DNA, *TCA* Tricarboxylic acid, *ROS* Reactive oxygen species

## Electronic supplementary material

Below is the link to the electronic supplementary material.

ESM 1 (DOCX 404 kb)
