# Supplementary material for: The pleiotropic effects of decanoic acid treatment on mitochondrial function in fibroblasts from patients with complex I deficient Leigh syndrome
Source: J Inherit Metab Dis. 2016 Apr 14;39(3):415–26. doi: 10.1007/s10545-016-9930-4 (PMC4851692; doi:10.1007/s10545-016-9930-4)
Supplement: Supplementary file 1 — (DOCX 404 kb) [file 10545_2016_9930_MOESM1_ESM.docx]

**Supplementary Information**

**The pleiotropic effects of decanoic acid treatment on mitochondrial function in fibroblasts from patients with complex I deficient Leigh syndrome**

Marta Kanabus^1^, Elisa Fassone^1^, Sean David Hughes^1,2^, Sara Farahi Bilooei^1^, Tricia Rutherford^3^, Maura O’ Donnell^3^, Simon J R Heales^1,2,4^, Shamima Rahman,^1,5^

^1^Genetics and Genomic Medicine, UCL Institute of Child Health, London WC1N 1EH, UK

^2^Chemical Pathology, Great Ormond Street Hospital for Children NHS Foundation Trust, London WC1N 3JH, UK

^3^Vitaflo International, Liverpool, UK

^4^Neurometabolic Unit, National Hospital for Neurology and Neurosurgery, London WC1N 3BG, UK

^5^Metabolic Department, Great Ormond Street Hospital Foundation Trust, London WC1N 3JH, UK

Corresponding Author:

Prof Shamima Rahman

UCL Institute of Child Health

30 Guilford Street

London WC1N 1EH

UK

Email: [shamima.rahman@ucl.ac.uk](mailto:shamima.rahman@ucl.ac.uk)

Tel: 020 79052608

Fax: 020 74046191

**Supplementary Methods**

*Citrate Synthase Enzyme Assay*

CS activity was measured in a 96-well plate (Corning) on the Tecan 200 Pro plate reader. 190 µl of CS buffer (100 mM Tris, 0.1% Triton X-100; pH adjusted to 8.0 with concentrated HCl) maintained at 37⁰C was pipetted into the 96-well plate, followed by 2 µl 10 mM DTNB (Sigma) in CS buffer, 2 µl of 5mM Acetyl CoA (Sigma) in CS buffer and 2 µl of 2 mg/ml of sample. The 96- well plate was kept on the plate reader drawer which was warmed up to 37⁰C at all times. An initial baseline reading was obtained by measuring absorbance for 2 min at 30 second intervals. Upon obtaining a baseline absorbance, 4 µl of 20 mM oxaloacetate (Sigma) in CS buffer was promptly added to each well using a multichannel pipette to reduce the time delay between samples. The absorbance was read again for 6 minutes at 30 second intervals. All reagents except for CS buffer were kept on ice. Special care was taken to work quickly and accurately. All samples had their CS levels measured in at least triplicate.

**Table S1** List of primers used for qPCR

| Gene Symbol | Gene Name | 5’-3’ Forward Primer Sequence | 5’-3’ Reverse Primer Sequence |
| --- | --- | --- | --- |
| *CS* | Citrate Synthase | GATGCTTGTCTTACTTGGCTGTT | AAGAACAAGACAAGATGCATTCC |
| *PPARG* | Peroxisome proliferator-activated receptor gamma | TTGCTGTCATTATTCTCAGTGGA | GAGGACTCAGGGTGGTTCAG |
| *PPARGC1A* | Peroxisome proliferator-activated receptor gamma co-activator 1-alpha | CGCAGTCACAACACTTACAAGC | CTTGGGGTCATTTGGTGACT |
| *NRF1* | Nuclear receptor 1 | GTAGTGCCTGGGTCCATGA | CCATCTGGTGGCCTGAAG |
| *NRF2* | Nuclear receptor 2 | GAGAGCCCAGTCTTCATTGC | TTGGCTTCTGGACTTGGAAC |
| *ERRA* | Estrogen receptor alpha | CCACTATGGTGTGGCATCCTGT | GGTGATCTCACACTCGTTGGAG |
| *ACADVL* | Acyl-CoA dehydrogenase, very long | ACCCGTCCGTGCTCAACGAA | CCAAGTGGTCTCCTCCACCAT |
| *HMOX1* | Haem oxygenase | CAGTCAGGCAGAGGGTGATAG | AGCTCCTGCAACTCCTCAAA |
| *CAT* | Catalase | GCTCATTTTGACCGAGAGAGA | TGACCTCAAAGTAGCCAAAGG |
| *TXNIP* | Thioredoxin interacting protein | GATCTGAACATCCCTGATACCC | AGAGGAGTGGTTGGGCTCTC |
| *B2M* | Beta-2 microglobulin | TGCTGTCTCCATGTTTGATGTATCT | TCTCTGCTCCCCACCTCTAAGT |
| *ACTB* | Beta actin | ATGACCCAGATCATGTTTGA | AGAGGCGTACAGGGATAG |

**Supplementary Results**

**Figure S1** Treatment of fibroblasts with C10 over a 6 day period increased CS activity in cells from 2 controls and 3 patients. Treatment of fibroblasts with BHB for 6 days at 5mM increased CS activity in cells from 2 patients (Patient 2 and 6). Treatment with ACA has no effect on CS activity. *p<0.05, **p<0.01

Key: ACA = acetoacetate, BHB = beta-hydroxybutyrate, C10 = decanoic acid, CS = citrate synthase, DMSO = dimethyl sulphoxide

A


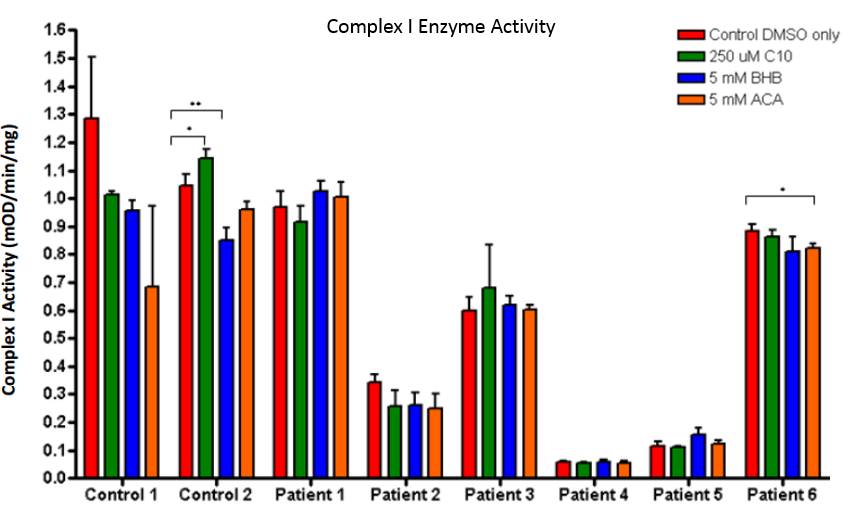


B


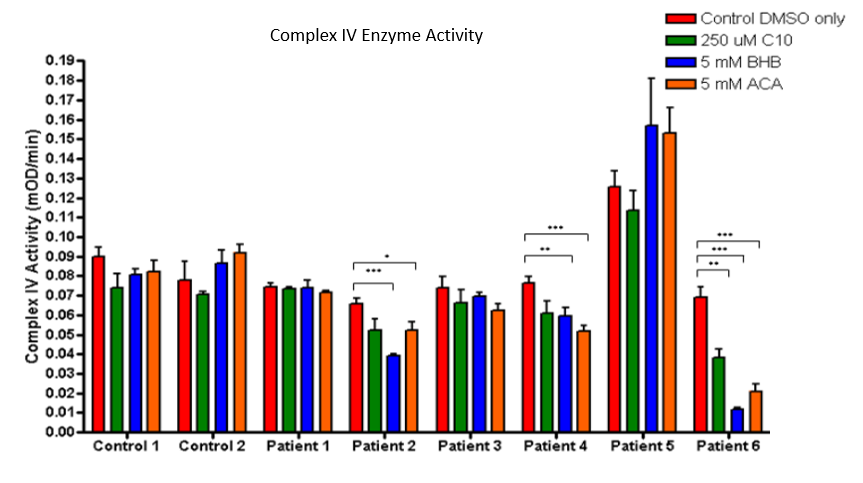


**Figure S2 A** -Complex I Activity was not affected by treatment with C10; **B -**Complex IV activity was decreased in one control upon a 6 day treatment with C10. *p<0.05

***

**

*

***

**Figure S3** Lactic Acid production was not affected by treatment with C10. Fibroblasts from four patients had increased baseline lactic acid production compared to control A. *p<0.05, **p<0.01, ***p<0.001


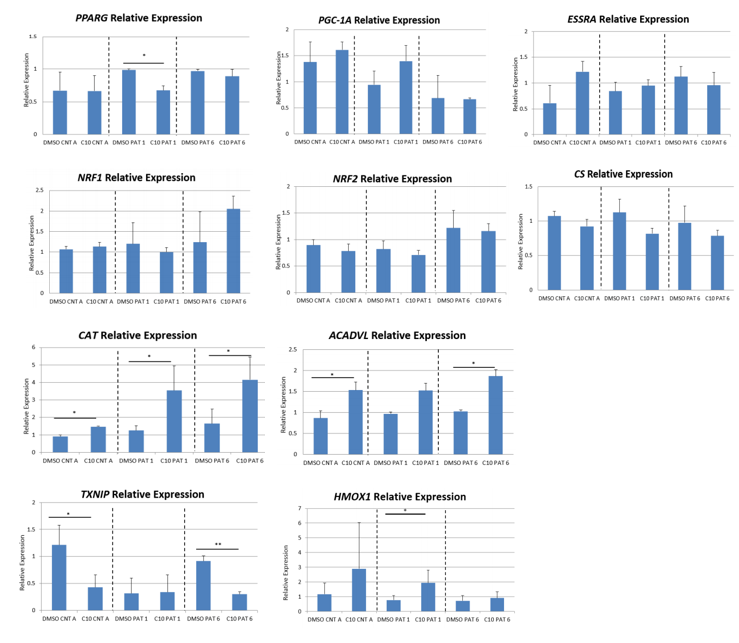


**Figure S4** qPCR results

**Table S2** An overview of fibroblast responses when treated with C10.


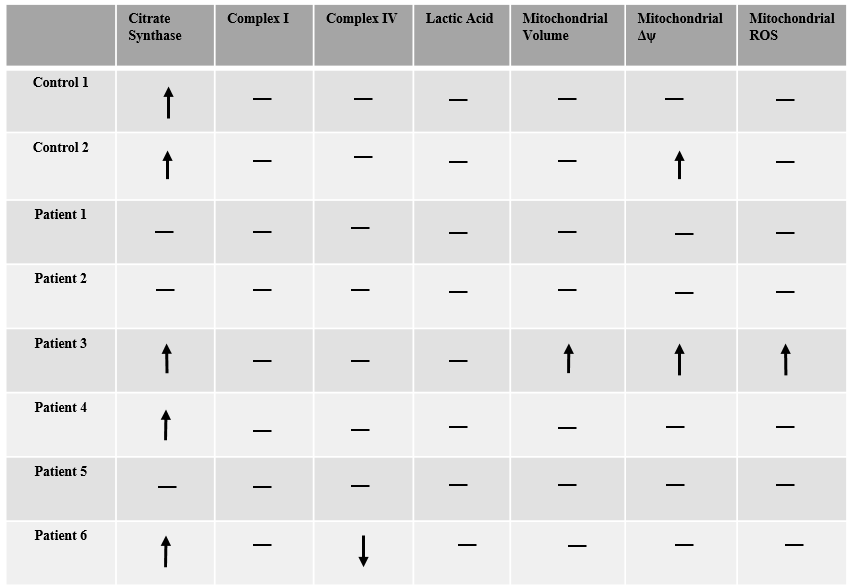


Key: 🡩 increased, 🡫 decreased, ̶ no change

**Table S3** Complete list of genes with increased and decreased expression upon treatment with C10

| Genes with decreased expression after C10 treatment | | | | |
| --- | --- | --- | --- | --- |
| Transcripts Cluster ID | **p - value** | **Fold Change** | **Gene** | **Gene Description** |
| 16681827 | 7.06E-06 | 3.161043 | *DHRS3* | dehydrogenase/reductase (SDR family) member 3 |
| 16669796 | 1.18E-07 | 2.46911 | *TXNIP* | thioredoxin interacting protein |
| 16718670 | 7.48E-07 | 2.425636 | *GFRA1* | GDNF family receptor alpha 1 |
| 16716718 | 2.46E-06 | 2.354777 | *LOC100128054* |  |
| 16754646 | 1.89E-05 | 2.229147 | *PTPRQ* | protein tyrosine phosphatase, receptor type, Q |
| 17045897 | 6.85E-05 | 2.218433 | *C7orf69* | chromosome 7 open reading frame 69 |
| 16970118 | 1.58E-06 | 2.201791 | *SYNPO2* | synaptopodin 2 |
| 16697471 | 8.01E-06 | 2.177154 | *B3GALT2* | UDP-Gal:betaGlcNAc beta 1,3-galactosyltransferase, polypeptide 2 |
| 16677556 | 5.48E-08 | 2.160673 | *TGFB2* | transforming growth factor, beta 2 |
| 17051553 | 1.65E-06 | 2.082175 | *CPA4* | carboxypeptidase A4 |
| 17069816 | 8.62E-08 | 2.031245 | *SULF1* | sulfatase 1 |
| 16844663 | 1.44E-05 | 2.028493 | *KRT34* | keratin 34 |
| 16858137 | 3.64E-05 | 1.972227 | *ICAM1* | intercellular adhesion molecule 1 |
| 16850724 | 3.25E-06 | 1.911308 | *ARHGAP28* | Rho GTPase activating protein 28 |
| 16803754 | 1.51E-05 | 1.877233 | *KIAA1199* |  |
| 16668220 | 6.96E-06 | 1.836331 | *SYPL2* | synaptophysin-like 2 |
| 16705961 | 5.03E-06 | 1.834488 | *DDIT4* | DNA-damage-inducible transcript 4 |
| 16698234 | 1.03E-05 | 1.820981 | *FMOD* | fibromodulin |
| 16908897 | 8.84E-06 | 1.819451 | *EPHA4* | EPH receptor A4 |
| 16690908 | 5.65E-08 | 1.818897 | *KCND3* | potassium voltage-gated channel, Shal-related subfamily, member 3 |
| 16743091 | 4.29E-06 | 1.817515 | *FZD4* | frizzled homolog 4 (Drosophila) |
| 16662047 | 1.51E-04 | 1.802707 | *FAM167B* | family with sequence similarity 167, member B |
| 17088100 | 1.42E-06 | 1.800582 | *UGCG* | UDP-glucose ceramide glucosyltransferase |
| 17062127 | 7.16E-05 | 1.799741 | *WNT2* | wingless-type MMTV integration site family member 2 |
| 16853399 | 2.52E-04 | 1.786766 | *COLEC12* | collectin sub-family member 12 |
| 16707196 | 7.72E-04 | 1.770139 | *IFIT1* | interferon-induced protein with tetratricopeptide repeats 1 |
| 16666055 | 5.69E-05 | 1.716325 | *CTH* | cystathionase (cystathionine gamma-lyase) |
| 16721884 | 1.82E-04 | 1.709079 | *LOC283104* |  |
| 16715228 | 1.66E-06 | 1.707311 | *C10orf54* | chromosome 10 open reading frame 54 |
| 16785086 | 1.20E-04 | 1.705222 | *SNAPC1* | small nuclear RNA activating complex, polypeptide 1, 43kDa |
| 17094893 | 1.55E-05 | 1.689974 | *ALDH1A1* | aldehyde dehydrogenase 1 family, member A1 |
| 16661862 | 5.21E-06 | 1.689519 | *TINAGL1* | tubulointerstitial nephritis antigen-like 1 |
| 17070013 | 1.19E-07 | 1.679849 | *RDH10* | retinol dehydrogenase 10 (all-trans) |
| 16788657 | 1.61E-04 | 1.678803 | *SNORD114-11* | small nucleolar RNA, C/D box 114-11 |
| 16970140 | 2.28E-07 | 1.678705 | *USP53* | ubiquitin specific peptidase 53 |
| 16937137 | 5.08E-05 | 1.674048 | *LMCD1* | LIM and cysteine-rich domains 1 |
| 16754808 | 1.82E-04 | 1.669164 | *TMTC2* | transmembrane and tetratricopeptide repeat containing 2 |
| 16894824 | 8.84E-07 | 1.668199 | *OSR1* | odd-skipped related 1 (Drosophila) |
| 17023799 | 1.59E-04 | 1.663569 | *SLC2A12* | solute carrier family 2 (facilitated glucose transporter), member 12 |
| 16788620 | 0.011293 | 1.65762 | *SNORD114-3* | small nucleolar RNA, C/D box 114-3 |
| 16917373 | 2.07E-04 | 1.653498 | *FLRT3* | fibronectin leucine rich transmembrane protein 3 |
| 16761820 | 7.65E-06 | 1.650207 | *MGP* | matrix Gla protein |
| 16676167 | 2.58E-05 | 1.647878 | *PRELP* | proline/arginine-rich end leucine-rich repeat protein |
| 16858118 | 0.013451 | 1.621368 | *SNORD105* | small nucleolar RNA, C/D box 105 |
| 16947173 | 2.83E-06 | 1.616221 | *MME* | membrane metallo-endopeptidase |
| 17095499 | 4.70E-06 | 1.607608 | *GAS1* | growth arrest-specific 1 |
| 16997275 | 5.43E-05 | 1.605227 | *GCNT4* | glucosaminyl (N-acetyl) transferase 4, core 2 |
| 16919962 | 9.14E-05 | 1.601434 | *SULF2* | sulfatase 2 |
| 16924620 | 1.13E-07 | 1.594281 | *ADAMTS5* | ADAM metallopeptidase with thrombospondin type 1 motif, 5 |
| 17013507 | 2.51E-05 | 1.592116 | *SAMD5* | sterile alpha motif domain containing 5 |
| 16667516 | 6.00E-05 | 1.591686 | *LPPR4* | lipid phosphate phosphatase-related protein type 4 |
| 17090296 | 9.85E-06 | 1.591459 | *ASS1* | argininosuccinate synthase 1 |
| 17086193 | 1.30E-06 | 1.590166 | *PSAT1* | phosphoserine aminotransferase 1 |
| 16880669 | 1.39E-08 | 1.586854 | *HSPC159* | galectin-related protein |
| 16760953 | 3.55E-05 | 1.586652 | *MFAP5* | microfibrillar associated protein 5 |
| 17046586 | 0.023846 | 1.583087 | *SNORA22* | small nucleolar RNA, H/ACA box 22 |
| 16821467 | 1.79E-04 | 1.580892 | *WFDC1* | WAP four-disulfide core domain 1 |
| 17012148 | 0.001994 | 1.578991 | *GJA1* | gap junction protein, alpha 1, 43kDa |
| 16767991 | 1.36E-05 | 1.578865 | *LIN7A* | lin-7 homolog A (C. elegans) |
| 17080486 | 1.16E-05 | 1.57564 | *TNFRSF11B* | tumor necrosis factor receptor superfamily, member 11b |
| 16690704 | 3.30E-04 | 1.57324 | *SLC16A4* | solute carrier family 16, member 4 (monocarboxylic acid transporter 5) |
| 16825371 | 4.69E-05 | 1.572003 | *NUPR1* | nuclear protein, transcriptional regulator, 1 |
| 16976644 | 2.31E-05 | 1.566004 | *IGJ* | immunoglobulin J polypeptide, linker protein for immunoglobulin alpha and mu polypeptides |
| 17051626 | 4.22E-05 | 1.563551 | *MEST* | mesoderm specific transcript homolog (mouse) |
| 16979339 | 1.98E-05 | 1.562579 | *PDE5A* | phosphodiesterase 5A, cGMP-specific |
| 16980974 | 2.03E-05 | 1.557082 | *FAM198B\|C4orf18* | family with sequence similarity 198, member B |
| 16768738 | 6.30E-06 | 1.545621 | *NTN4* | netrin 4 |
| 16743816 | 2.22E-04 | 1.542954 | *PDGFD* | platelet derived growth factor D |
| 17057478 | 5.99E-07 | 1.542057 | *IGFBP3* | insulin-like growth factor binding protein 3 |
| 17012281 | 3.04E-05 | 1.536478 | *TPD52L1* | tumor protein D52-like 1 |
| 16941024 | 1.06E-05 | 1.523923 | *SEMA3B* | sema domain, immunoglobulin domain (Ig), short basic domain, secreted, (semaphorin) 3B |
| 17013126 | 5.76E-05 | 1.519839 | *GPR126* | G protein-coupled receptor 126 |
| 16788653 | 0.007547 | 1.516983 | *SNORD114-9* | small nucleolar RNA, C/D box 114-9 |
| 16705507 | 6.20E-04 | 1.511732 | *SRGN* | serglycin |
| 16793129 | 1.53E-06 | 1.507392 | *BMP4* | bone morphogenetic protein 4 |
| 16768413 | 3.84E-04 | 1.50562 | *DCN* | decorin |
| 16853807 | 7.30E-05 | 1.504616 | *LOC100287082* |  |
| 16748529 | 1.42E-05 | 1.501915 | *GPRC5A* | G protein-coupled receptor, family C, group 5, member A |
| 17078254 | 4.79E-05 | 1.497871 | *C8orf84* | chromosome 8 open reading frame 84 |
| 16997399 | 0.015156 | 1.495747 | *SNORA47* | small nucleolar RNA, H/ACA box 47 |
| 16968213 | 1.49E-07 | 1.494942 | *ANXA3* | annexin A3 |
| 16945768 | 2.10E-05 | 1.490521 | *CCRL1* | chemokine (C-C motif) receptor-like 1 |
| 16756310 | 2.67E-04 | 1.489213 | *TCP11L2* | t-complex 11 (mouse)-like 2 |
| 16938407 | 1.14E-06 | 1.485076 | *RARB* | retinoic acid receptor, beta |
| 16897834 | 7.98E-04 | 1.483002 | *EFEMP1* | EGF-containing fibulin-like extracellular matrix protein 1 |
| 16698801 | 6.86E-04 | 1.479873 | *CD34* |  |
| 16858120 | 0.002098 | 1.477392 | *SNORD105B* | small nucleolar RNA, C/D box 105B |
| 17060061 | 6.40E-06 | 1.474975 | *ASNS* | asparagine synthetase (glutamine-hydrolyzing) |
| 17095887 | 0.005058 | 1.474873 | *ASPN* | asporin |
| 16774303 | 9.53E-04 | 1.473478 | *C13orf15* | chromosome 13 open reading frame 15 |
| 17094761 | 8.23E-06 | 1.471132 | *KLF9* | Kruppel-like factor 9 |
| 16751655 | 0.001058 | 1.466373 | *IGFBP6* | insulin-like growth factor binding protein 6 |
| 16673748 | 1.03E-04 | 1.466143 | *FMO4* | flavin containing monooxygenase 4 |
| 16760691 | 1.74E-04 | 1.465509 | *C1R* | complement component 1, r subcomponent |
| 17076861 | 6.76E-06 | 1.457954 | *CEBPD* | CCAAT/enhancer binding protein (C/EBP), delta |
| 16938875 | 0.004363 | 1.453483 | *STAC* | SH3 and cysteine rich domain |
| 16788616 | 0.007768 | 1.453363 | *SNORD114-1* | small nucleolar RNA, C/D box 114-1 |
| 16705188 | 6.99E-04 | 1.451407 | *C10orf107* | chromosome 10 open reading frame 107 |
| 16954217 | 2.38E-04 | 1.431725 | *UBA7* | ubiquitin-like modifier activating enzyme 7 |
| 16761293 | 2.17E-04 | 1.431047 | *OLR1* | oxidized low density lipoprotein (lectin-like) receptor 1 |
| 17085760 | 0.001795 | 1.42295 | *MAMDC2* | MAM domain containing 2 |
| 16769481 | 5.38E-04 | 1.421055 | *ALDH1L2* | aldehyde dehydrogenase 1 family, member L2 |
| 16947357 | 2.07E-04 | 1.420576 | *PTX3* | pentraxin 3, long |
| 16765945 | 1.99E-05 | 1.419828 | *RNF41* | ring finger protein 41 |
| 16672214 | 2.76E-04 | 1.414092 | *PEAR1* | platelet endothelial aggregation receptor 1 |
| 16686201 | 1.41E-05 | 1.412223 | *SLC6A9* | solute carrier family 6 (neurotransmitter transporter, glycine), member 9 |
| 16670894 | 1.56E-05 | 1.411473 | *TUFT1* | tuftelin 1 |
| 16977502 | 2.05E-06 | 1.410247 | *PLAC8* | placenta-specific 8 |
| 17050350 | 2.47E-06 | 1.405508 | *LRRN3* | leucine rich repeat neuronal 3 |
| 16926111 | 4.77E-07 | 1.404958 | *CBS* | cystathionine-beta-synthase |
| 16668997 | 5.41E-04 | 1.402104 | *OLFML3* | olfactomedin-like 3 |
| 17002052 | 9.65E-06 | 1.40162 | *C5orf4* | chromosome 5 open reading frame 4 |
| 16987395 | 6.40E-07 | 1.401136 | *RHOBTB3* | Rho-related BTB domain containing 3 |
| 16979985 | 2.77E-06 | 1.395297 | *C4orf49\|NDUFC1* | chromosome 4 open reading frame 49 \| NADH dehydrogenase (ubiquinone) 1, subcomplex unknown, 1, 6kDa |
| 17006066 | 5.59E-04 | 1.393686 | *SNORD32B* | small nucleolar RNA, C/D box 32B |
| 16842659 | 3.64E-05 | 1.386817 | *ALDOC* | aldolase C, fructose-bisphosphate |
| 17069550 | 7.44E-05 | 1.386238 | *ADHFE1* | alcohol dehydrogenase, iron containing, 1 |
| 16979845 | 0.004069 | 1.384666 | *KRT18* | keratin 18 |
| 16747678 | 0.002277 | 1.383189 | *C1S* | complement component 1, s subcomponent |
| 16875518 | 4.43E-04 | 1.382737 | *CDC42EP5* | CDC42 effector protein (Rho GTPase binding) 5 |
| 16838330 | 0.001781 | 1.38247 | *SYNGR2* | synaptogyrin 2 |
| 17004903 | 2.94E-05 | 1.381519 | *EDN1* | endothelin 1 |
| 16877616 | 4.58E-05 | 1.380949 | *KLHL29* | kelch-like 29 (Drosophila) |
| 17083197 | 2.47E-04 | 1.380568 | *VLDLR* | very low density lipoprotein receptor |
| 17063977 | 2.33E-04 | 1.379541 | *LOC154761* |  |
| 16773493 | 2.65E-05 | 1.375366 | *RASL11A* | RAS-like, family 11, member A |
| 16992467 | 2.39E-04 | 1.374304 | *C5orf41* | chromosome 5 open reading frame 41 |
| 16988133 | 2.48E-05 | 1.373146 | *KCNN2* | potassium intermediate/small conductance calcium-activated channel, subfamily N, member 2 |
| 16731461 | 7.11E-04 | 1.372966 | *NNMT* | nicotinamide N-methyltransferase |
| 16882285 | 9.72E-05 | 1.367711 | *VAMP5* | vesicle-associated membrane protein 5 (myobrevin) |
| 16700400 | 1.09E-04 | 1.366307 | *C1orf198* | chromosome 1 open reading frame 198 |
| 16762154 | 0.002012 | 1.363947 | *ABCC9* | ATP-binding cassette, sub-family C (CFTR/MRP), member 9 |
| 16892826 | 3.77E-04 | 1.363748 | *CXCR7* | chemokine (C-X-C motif) receptor 7 |
| 16867715 | 4.27E-04 | 1.363209 | *SLC25A23* | solute carrier family 25 (mitochondrial carrier; phosphate carrier), member 23 |
| 16723058 | 1.38E-05 | 1.36236 | *FIBIN* | fin bud initiation factor homolog (zebrafish) |
| 16825638 | 8.95E-04 | 1.360061 | *YPEL3* | yippee-like 3 (Drosophila) |
| 17025619 | 2.81E-04 | 1.359844 | *RPS6KA2* | ribosomal protein S6 kinase, 90kDa, polypeptide 2 |
| 16763295 | 4.41E-04 | 1.359368 | *PRICKLE1* | prickle homolog 1 (Drosophila) |
| 17080516 | 0.010916 | 1.3569 | *ENPP2* | ectonucleotide pyrophosphatase/phosphodiesterase 2 |
| 17062280 | 1.06E-04 | 1.355667 | *AASS* | aminoadipate-semialdehyde synthase |
| 16684080 | 6.33E-04 | 1.352994 | *IFI6* | interferon, alpha-inducible protein 6 |
| 16948572 | 5.02E-04 | 1.351779 | *KLHL24* | kelch-like 24 (Drosophila) |
| 16881660 | 1.57E-05 | 1.35142 | *MTHFD2* | methylenetetrahydrofolate dehydrogenase (NADP+ dependent) 2, methenyltetrahydrofolate cyclohydrolase |
| 16782548 | 2.74E-04 | 1.34962 | *PCK2* | phosphoenolpyruvate carboxykinase 2 (mitochondrial) |
| 17005077 | 0.006155 | 1.348618 | *MYLIP* | myosin regulatory light chain interacting protein |
| 17071144 | 3.56E-05 | 1.346524 | *SDC2* | syndecan 2 |
| 16796694 | 6.35E-05 | 1.345532 | *WARS* | tryptophanyl-tRNA synthetase |
| 16674973 | 4.03E-05 | 1.345274 | *C1orf21* | chromosome 1 open reading frame 21 |
| 16660360 | 1.49E-04 | 1.345087 | *CDA* | cytidine deaminase |
| 16755173 | 6.07E-04 | 1.344892 | *PLXNC1* | plexin C1 |
| 17071298 | 5.62E-05 | 1.344811 | *OSR2* | odd-skipped related 2 (Drosophila) |
| 17013084 | 0.003486 | 1.343249 | *LOC100132735* |  |
| 17060167 | 0.00102 | 1.342989 | *TMEM130* | transmembrane protein 130 |
| 16689384 | 2.43E-04 | 1.342555 | *GBP4* | guanylate binding protein 4 |
| 17002612 | 1.42E-04 | 1.342489 | *SLIT3* | slit homolog 3 (Drosophila) |
| 16677071 | 1.54E-04 | 1.341036 | *SERTAD4* | SERTA domain containing 4 |
| 16791455 | 1.40E-04 | 1.338933 | *STXBP6* | syntaxin binding protein 6 (amisyn) |
| 16675398 | 0.023849 | 1.338114 | *CFH* | complement factor H |
| 17057946 | 4.00E-04 | 1.334745 | *PSPH* | phosphoserine phosphatase |
| 16801299 | 0.008027 | 1.333127 | *FLJ27352* | hypothetical LOC145788 |
| 16775763 | 0.003385 | 1.328772 | *MIR622* | microRNA 622 |
| 16755908 | 2.27E-05 | 1.328727 | *DRAM1* | DNA-damage regulated autophagy modulator 1 |
| 16917183 | 9.77E-04 | 1.328303 | *JAG1* | jagged 1 |
| 16811085 | 0.001314 | 1.328126 | *ITGA11* | integrin, alpha 11 |
| 17056426 | 1.19E-04 | 1.327756 | *PDE1C* | phosphodiesterase 1C, calmodulin-dependent 70kDa |
| 17016221 | 5.21E-06 | 1.324669 | *FAM65B* | family with sequence similarity 65, member B |
| 16855973 | 4.68E-05 | 1.32368 | *CYB5A* | cytochrome b5 type A (microsomal) |
| 17010522 | 0.001239 | 1.323545 | *IRAK1BP1* | interleukin-1 receptor-associated kinase 1 binding protein 1 |
| 16908197 | 8.75E-05 | 1.320831 | *IGFBP5* | insulin-like growth factor binding protein 5 |
| 16904667 | 3.63E-04 | 1.3199 | *SCN9A* | sodium channel, voltage-gated, type IX, alpha subunit |
| 17106438 | 1.42E-04 | 1.319598 | *DOCK11* | dedicator of cytokinesis 11 |
| 16775811 | 6.94E-04 | 1.318475 | *GPC6* | glypican 6 |
| 16910609 | 6.42E-05 | 1.318302 | *TRIB3* | tribbles homolog 3 (Drosophila) |
| 16851565 | 1.05E-05 | 1.318166 | *TTC39C* | tetratricopeptide repeat domain 39C |
| 16829570 | 0.012238 | 1.317575 | *SERPINF1* | serpin peptidase inhibitor, clade F (alpha-2 antiplasmin, pigment epithelium derived factor), member 1 |
| 16715361 | 0.005898 | 1.317081 | *P4HA1* | prolyl 4-hydroxylase, alpha polypeptide I |
| 16958403 | 1.88E-04 | 1.317053 | *HEG1* | HEG homolog 1 (zebrafish) |
| 17050361 | 4.21E-04 | 1.316285 | *ZNF277* | zinc finger protein 277 |
| 16712773 | 2.44E-04 | 1.314183 | *MKX* | mohawk homeobox |
| 16911651 | 2.41E-04 | 1.3135 | *DSTN* | destrin (actin depolymerizing factor) |
| 16764894 | 0.003161 | 1.313433 | *KRT6B* | keratin 6B |
| 16713530 | 1.21E-05 | 1.313346 | *CXCL12* | chemokine (C-X-C motif) ligand 12 |
| 16931237 | 2.02E-04 | 1.313169 | *FBLN1* | fibulin 1 |
| 16800962 | 0.002672 | 1.3129 | *SLC27A2* | solute carrier family 27 (fatty acid transporter), member 2 |
| 16719587 | 0.007493 | 1.312824 | *MIR4297* | microRNA 4297 |
| 16768406 | 0.001636 | 1.311864 | *LUM* | lumican |
| 16872944 | 0.009845 | 1.309657 | *PSG4* | pregnancy specific beta-1-glycoprotein 4 |
| 16878994 | 0.001609 | 1.309339 | *RASGRP3* | RAS guanyl releasing protein 3 (calcium and DAG-regulated) |
| 16721905 | 7.38E-06 | 1.308783 | *ADM* | adrenomedullin |
| 16937152 | 0.035158 | 1.308623 | *LOH3CR2A* | loss of heterozygosity, 3, chromosomal region 2, gene A |
| 17069886 | 2.46E-04 | 1.307437 | *TRAM1* | translocation associated membrane protein 1 |
| 16879500 | 5.08E-04 | 1.307372 | *PLEKHH2* | pleckstrin homology domain containing, family H (with MyTH4 domain) member 2 |
| 16720085 | 8.30E-04 | 1.306712 | *IFITM1* | interferon induced transmembrane protein 1 (9-27) |
| 16885290 | 1.04E-06 | 1.30645 | *GYPC* | glycophorin C (Gerbich blood group) |
| 16816579 | 8.59E-05 | 1.304457 | *ACSM2A* | acyl-CoA synthetase medium-chain family member 2A |
| 16744572 | 0.027937 | 1.304065 | *LOC100506941* |  |
| 16769569 | 4.41E-04 | 1.303512 | *NUAK1* | NUAK family, SNF1-like kinase, 1 |
| 17077502 | 1.02E-04 | 1.303113 | *TOX* | thymocyte selection-associated high mobility group box |
| 17012207 | 0.004373 | 1.302932 | *SMPDL3A* | sphingomyelin phosphodiesterase, acid-like 3A |
| 16799739 | 6.54E-05 | 1.302828 | *CHAC1* | ChaC, cation transport regulator homolog 1 (E. coli) |
| 17056227 | 7.53E-05 | 1.302823 | *JAZF1* | JAZF zinc finger 1 |

| Genes with increased expression after C10 treatment | | | | |  |
| --- | --- | --- | --- | --- | --- |
| Transcripts Cluster ID | **p - value** | **Fold Change** | **Gene** | **Gene Description** |  |
| 16743721 | 3.65E-05 | 3.1424558 | *MMP1* | matrix metallopeptidase 1 (interstitial collagenase) | |
| 16857886 | 9.34E-09 | 2.7146022 | *ANGPTL4* | angiopoietin-like 4 |  |
| 16984689 | 2.64E-06 | 2.5721283 | *ITGA2* | integrin, alpha 2 (CD49B, alpha 2 subunit of VLA-2 receptor) | |
| 17110365 | 1.20E-05 | 2.369402 | *MIR221* | microRNA 221 |  |
| 17080648 | 4.20E-05 | 2.236638 | *HAS2* | hyaluronan synthase 2 |  |
| 17097661 | 7.12E-06 | 2.1926312 | *TNC* | tenascin C |  |
| 16767851 | 2.12E-06 | 2.093546 | *E2F7* | E2F transcription factor 7 |  |
| 17079210 | 4.35E-06 | 2.0145648 | *GEM* | GTP binding protein overexpressed in skeletal muscle | |
| 16901497 | 3.65E-07 | 1.9891797 | *ST6GAL2* | ST6 beta-galactosamide alpha-2,6-sialyltranferase 2 | |
| 17113448 | 1.80E-04 | 1.9317281 | *IL13RA2* | interleukin 13 receptor, alpha 2 | |
| 17104519 | 2.73E-04 | 1.9146681 | *RNY4P23* |  |  |
| 17110369 | 6.73E-06 | 1.8893907 |  |  |  |
| 17092870 | 5.11E-07 | 1.8589097 | *LOC554202* |  |  |
| 17012632 | 4.68E-06 | 1.8527352 | *ENPP1* | ectonucleotide pyrophosphatase/phosphodiesterase 1 | |
| 16978568 | 5.56E-06 | 1.8218762 | *CENPE* | centromere protein E, 312kDa | |
| 16887702 | 7.70E-05 | 1.8039645 | *ITGA6* | integrin, alpha 6 |  |
| 16952782 | 2.40E-06 | 1.770664 | *TMEM158* | transmembrane protein 158 (gene/pseudogene) | |
| 16996146 | 1.59E-04 | 1.7512085 | *ESM1* | endothelial cell-specific molecule 1 | |
| 16784137 | 0.041148 | 1.747259 |  |  |  |
| 16691883 | 3.02E-04 | 1.7441771 | *FAM72D* | family with sequence similarity 72, member D | |
| 16741287 | 1.14E-05 | 1.7420118 | *CPT1A* | carnitine palmitoyltransferase 1A (liver) | |
| 16798810 | 0.001253 | 1.7377415 |  |  |  |
| 17002667 | 4.56E-06 | 1.7263925 | *FAM196B* | family with sequence similarity 196, member B | |
| 16677425 | 3.35E-06 | 1.695235 | *CENPF* | centromere protein F, 350/400kDa (mitosin) | |
| 17058826 | 7.61E-08 | 1.693401 | *HIP1* | huntingtin interacting protein 1 | |
| 16677201 | 2.93E-06 | 1.6923984 | *DTL* | denticleless homolog (Drosophila) | |
| 16943230 | 3.69E-06 | 1.6852599 |  |  |  |
| 17070221 | 5.84E-05 | 1.6712785 | *PKIA* | protein kinase (cAMP-dependent, catalytic) inhibitor alpha | |
| 16767115 | 5.10E-06 | 1.6698985 | *RPSAP52* | ribosomal protein SA pseudogene 52 | |
| 16719515 | 7.13E-05 | 1.6678184 | *MKI67* | antigen identified by monoclonal antibody Ki-67 | |
| 16870925 | 6.20E-05 | 1.6666977 | *ZNF724P* | zinc finger protein 724, pseudogene | |
| 16696531 | 1.48E-04 | 1.6653602 | *SNORD78* |  |  |
| 17055354 | 4.77E-06 | 1.6649034 | *ETV1* | ets variant 1 |  |
| 17016403 | 4.38E-05 | 1.6610067 | *HIST1H3G* | histone cluster 1, H3g |  |
| 16702571 | 4.14E-05 | 1.6534903 | *MCM10* | minichromosome maintenance complex component 10 | |
| 17011005 | 0.013538 | 1.6503893 |  |  |  |
| 17110372 | 9.51E-04 | 1.6496799 | *FLJ25917* | hypothetical LOC401585 |  |
| 16798696 | 6.82E-04 | 1.6467127 |  |  |  |
| 17092712 | 6.30E-08 | 1.6453522 | *PLIN2* | perilipin 2 |  |
| 17010760 | 4.02E-05 | 1.6440831 | *NT5E* | 5'-nucleotidase, ecto (CD73) |  |
| 16780069 | 3.11E-07 | 1.6407161 | *SPRY2* | sprouty homolog 2 (Drosophila) | |
| 16986913 | 2.79E-07 | 1.6377529 | *VCAN* | versican |  |
| 16882975 | 2.68E-06 | 1.6356418 | *NCAPH* | non-SMC condensin I complex, subunit H | |
| 16872419 | 0.005897 | 1.6316909 |  |  |  |
| 16753663 | 0.038129 | 1.6240282 |  |  |  |
| 16766627 | 1.98E-06 | 1.6211984 | *B4GALNT1* | beta-1,4-N-acetyl-galactosaminyl transferase 1 | |
| 16793999 | 2.77E-05 | 1.6210728 | *PLEK2* | pleckstrin 2 |  |
| 16842673 | 6.56E-06 | 1.618127 | *SPAG5\|SGK494* | sperm associated antigen 5 \| uncharacterized serine/threonine-protein kinase SgK494 | |
| 17116675 | 0.009494 | 1.6156696 |  |  |  |
| 17016400 | 7.63E-04 | 1.6145871 | *HIST1H3F* | histone cluster 1, H3f |  |
| 16970563 | 5.54E-06 | 1.614248 | *PLK4* | polo-like kinase 4 |  |
| 16852445 | 1.27E-06 | 1.6116118 | *C18orf54* | chromosome 18 open reading frame 54 | |
| 17062945 | 0.003332 | 1.6115229 | *LOC646329* |  |  |
| 17059955 | 3.91E-06 | 1.6098636 | *PDK4* | pyruvate dehydrogenase kinase, isozyme 4 | |
| 16824352 | 4.58E-06 | 1.6089965 | *XYLT1* | xylosyltransferase I |  |
| 17062941 | 0.001435 | 1.6087451 | *MIR29A* | microRNA 29a |  |
| 16740630 | 1.62E-06 | 1.606256 | *FOSL1* | FOS-like antigen 1 |  |
| 16992096 | 9.82E-06 | 1.6057596 | *CCDC99* | coiled-coil domain containing 99 | |
| 16745507 | 0.036737 | 1.6013947 | *MIRLET7A2* | microRNA let-7a-2 |  |
| 16798801 | 4.46E-06 | 1.6008494 | *ARHGAP11B* | Rho GTPase activating protein 11B | |
| 16901755 | 1.66E-05 | 1.6004947 | *BUB1* | budding uninhibited by benzimidazoles 1 homolog (yeast) | |
| 16826160 | 3.37E-05 | 1.6001518 | *SHCBP1* | SHC SH2-domain binding protein 1 | |
| 16847432 | 1.18E-05 | 1.5988721 | *BRIP1* | BRCA1 interacting protein C-terminal helicase 1 | |
| 16707468 | 1.33E-05 | 1.5977057 | *KIF11* | kinesin family member 11 |  |
| 16804631 | 7.47E-05 | 1.5972884 | *C15orf42* | chromosome 15 open reading frame 42 | |
| 16697695 | 3.40E-05 | 1.5970292 | *KIF14* | kinesin family member 14 |  |
| 16829764 | 1.65E-05 | 1.5870249 | *GSG2* | germ cell associated 2 (haspin) | |
| 16947603 | 0.003086 | 1.5856806 | *MIR15B* | microRNA 15b |  |
| 16707695 | 3.14E-04 | 1.5851642 | *HELLS* | helicase, lymphoid-specific |  |
| 16852312 | 1.26E-06 | 1.5791982 | *SKA1* | spindle and kinetochore associated complex subunit 1 | |
| 16799598 | 7.58E-06 | 1.5791788 | *CASC5* | cancer susceptibility candidate 5 | |
| 17016369 | 7.56E-05 | 1.5775857 | *HIST1H2BB* | histone cluster 1, H2bb |  |
| 16766403 | 1.18E-04 | 1.5761131 | *TMEM194A* | transmembrane protein 194A | |
| 16673652 | 0.043745 | 1.5727984 | *LOC100127910* |  |  |
| 16707221 | 1.36E-04 | 1.5716177 | *KIF20B* | kinesin family member 20B |  |
| 16995938 | 2.74E-04 | 1.5715716 | *C5orf34* | chromosome 5 open reading frame 34 | |
| 16920730 | 1.25E-06 | 1.5711972 | *APCDD1L* | adenomatosis polyposis coli down-regulated 1-like | |
| 17016383 | 4.58E-05 | 1.5696509 | *HIST1H4D* | histone cluster 1, H4d |  |
| 16798919 | 1.86E-06 | 1.5619364 | *ARHGAP11A* | Rho GTPase activating protein 11A | |
| 16996722 | 1.12E-05 | 1.5607426 | *CENPK* | centromere protein K |  |
| 16901957 | 1.38E-06 | 1.5583677 | *CKAP2L* | cytoskeleton associated protein 2-like | |
| 16692614 | 7.99E-04 | 1.558339 | *HIST2H3D* | histone cluster 2, H3d |  |
| 16815090 | 8.40E-05 | 1.552219 | *CCNF* | cyclin F |  |
| 16725041 | 3.91E-05 | 1.5518432 | *FAM111B* | family with sequence similarity 111, member B | |
| 16830302 | 1.80E-05 | 1.5470351 | *ACADVL* | acyl-CoA dehydrogenase, very long chain | |
| 17016496 | 0.002473 | 1.5441641 | *HIST1H2AK* | histone cluster 1, H2ak |  |
| 17067102 | 1.68E-06 | 1.5424724 | *CDCA2* | cell division cycle associated 2 | |
| 16773840 | 9.56E-05 | 1.5423157 | *BRCA2* | breast cancer 2, early onset |  |
| 17079910 | 5.64E-08 | 1.5410074 | *KLF10* | Kruppel-like factor 10 |  |
| 16957951 | 4.31E-04 | 1.5407181 | *POLQ* | polymerase (DNA directed), theta | |
| 16753641 | 0.003959 | 1.5358324 | *HMGA2* | high mobility group AT-hook 2 | |
| 16701037 | 5.37E-06 | 1.5354526 | *GREM2* | gremlin 2 |  |
| 16771067 | 1.20E-05 | 1.5297391 | *CIT\|MIR1178* | citron (rho-interacting, serine/threonine kinase 21) \| microRNA 1178 | |
| 16685165 | 2.19E-05 | 1.5264343 | *CLSPN* | claspin |  |
| 16914315 | 1.66E-05 | 1.5238105 | *UBE2C* | ubiquitin-conjugating enzyme E2C | |
| 16832852 | 9.66E-04 | 1.5228916 | *ATAD5* | ATPase family, AAA domain containing 5 | |
| 16711322 | 2.98E-04 | 1.5220416 |  |  |  |
| 16860103 | 1.34E-05 | 1.5199051 | *ZNF93\|ZNF90* | zinc finger protein 93 \| zinc finger protein 90 | |
| 16850517 | 6.90E-06 | 1.5189408 | *NDC80* |  |  |
| 17102566 | 1.95E-06 | 1.5183135 | *SYTL5* | synaptotagmin-like 5 |  |
| 16799635 | 0.001768 | 1.5179412 |  |  |  |
| 16852871 | 2.31E-04 | 1.5177075 | *SERPINB2\|SERPINB10* | serpin peptidase inhibitor, clade B (ovalbumin), member 2 \| serpin peptidase inhibitor, clade B (ovalbumin), member 10 | |
| 16877007 | 1.53E-07 | 1.517232 | *KLF11* | Kruppel-like factor 11 |  |
| 16743405 | 0.0124 | 1.5147494 |  |  |  |
| 17001063 | 4.60E-06 | 1.5121182 | *SPRY4* | sprouty homolog 4 (Drosophila) | |
| 16738630 | 1.78E-05 | 1.5113015 | *LPXN* | leupaxin |  |
| 16909700 | 5.73E-06 | 1.51113 | *HJURP* | Holliday junction recognition protein | |
| 16664243 | 6.22E-06 | 1.5106946 | *RAD54L* | RAD54-like (S. cerevisiae) |  |
| 16965346 | 8.98E-05 | 1.5101112 | *NCAPG* | non-SMC condensin I complex, subunit G | |
| 16728141 | 1.08E-05 | 1.5076376 | *GAL* | galanin prepropeptide |  |
| 16845794 | 4.63E-06 | 1.506615 | *KIF18B* | kinesin family member 18B |  |
| 16877473 | 1.85E-04 | 1.5062251 | *GEN1* | Gen homolog 1, endonuclease (Drosophila) | |
| 17079293 | 3.47E-05 | 1.5056294 | *CCNE2* | cyclin E2 |  |
| 16804559 | 5.19E-05 | 1.5055584 | *FANCI* | Fanconi anemia, complementation group I | |
| 17086359 | 0.005104 | 1.5038786 |  |  |  |
| 16697544 | 2.83E-05 | 1.5037009 | *ASPM* | asp (abnormal spindle) homolog, microcephaly associated (Drosophila) | |
| 16820820 | 0.046195 | 1.5035206 | *SNORD111* | small nucleolar RNA, C/D box 111 | |
| 16711413 | 9.49E-05 | 1.503024 |  |  |  |
| 16679411 | 1.76E-04 | 1.5019225 | *EXO1* | exonuclease 1 |  |
| 16951485 | 1.43E-05 | 1.5010104 | *SGOL1* | shugoshin-like 1 (S. pombe) |  |
| 17092688 | 9.40E-05 | 1.5002595 | *HAUS6* | HAUS augmin-like complex, subunit 6 | |
| 16826738 | 0.005821 | 1.5002592 | *MT1G* | metallothionein 1G |  |
| 16963667 | 0.049378 | 1.499497 |  |  |  |
| 16662648 | 3.69E-05 | 1.499414 | *CDCA8* | cell division cycle associated 8 | |
| 16911212 | 8.38E-04 | 1.4979941 | *MCM8* | minichromosome maintenance complex component 8 | |
| 16868838 | 3.30E-06 | 1.4970585 | *SPC24* |  |  |
| 16793190 | 1.96E-05 | 1.4931791 | *WDHD1* | WD repeat and HMG-box DNA binding protein 1 | |
| 17078870 | 1.90E-04 | 1.4899176 | *MMP16* | matrix metallopeptidase 16 (membrane-inserted) | |
| 16694617 | 8.79E-06 | 1.4893516 | *IQGAP3* | IQ motif containing GTPase activating protein 3 | |
| 16682702 | 0.009806 | 1.4890864 |  |  |  |
| 16991741 | 0.003586 | 1.4887518 | *PTTG1* | pituitary tumor-transforming 1 | |
| 16939960 | 1.84E-05 | 1.4880661 | *KIF15* | kinesin family member 15 |  |
| 16667175 | 0.005302 | 1.4870938 | *SNORA66* | small nucleolar RNA, H/ACA box 66 | |
| 16716657 | 0.002862 | 1.4868091 |  |  |  |
| 16813342 | 1.85E-05 | 1.4857883 | *PRC1* | protein regulator of cytokinesis 1 | |
| 16661429 | 1.13E-05 | 1.4846323 | *GPR3* | G protein-coupled receptor 3 | |
| 17016356 | 7.25E-05 | 1.4834557 | *HIST1H1A* | histone cluster 1, H1a |  |
| 17086784 | 1.72E-04 | 1.4834329 | *CENPP* | centromere protein P |  |
| 16985614 | 9.91E-05 | 1.4830601 | *CENPH* | centromere protein H |  |
| 16684192 | 0.004989 | 1.4810948 | *SNORD99* | small nucleolar RNA, C/D box 99 | |
| 16991460 | 7.85E-04 | 1.4794118 | *KIF4B* | kinesin family member 4B |  |
| 16931384 | 7.29E-05 | 1.4792619 | *GTSE1* | G-2 and S-phase expressed 1 | |
| 17047086 | 0.005484 | 1.4791743 | *MIR590* | microRNA 590 |  |
| 16884523 | 1.79E-05 | 1.4767619 | *SLC20A1* | solute carrier family 20 (phosphate transporter), member 1 | |
| 17064939 | 1.04E-05 | 1.4767599 | *NCAPG2* | non-SMC condensin II complex, subunit G2 | |
| 16736891 | 3.00E-04 | 1.4753203 | *KIF18A* | kinesin family member 18A |  |
| 17051441 | 2.34E-04 | 1.474805 | *FAM40B* | family with sequence similarity 40, member B | |
| 16698984 | 1.48E-04 | 1.4738803 | *NEK2* | NIMA (never in mitosis gene a)-related kinase 2 | |
| 16792519 | 5.75E-06 | 1.4726942 | *POLE2* | polymerase (DNA directed), epsilon 2 (p59 subunit) | |
| 17068782 | 1.35E-06 | 1.4725468 | *MCM4* | minichromosome maintenance complex component 4 | |
| 16800355 | 2.34E-05 | 1.4718072 | *WDR76* | WD repeat domain 76 |  |
| 16755498 | 4.24E-06 | 1.4717896 | *TMPO* | thymopoietin |  |
| 16726880 | 0.017544 | 1.4692419 | *NEAT1* |  |  |
| 16739636 | 0.001074 | 1.468003 | *SNHG1* | small nucleolar RNA host gene 1 (non-protein coding) | |
| 16957170 | 9.60E-05 | 1.4678255 | *KIAA1524* |  |  |
| 16919044 | 3.99E-05 | 1.4673768 | *RBL1* | retinoblastoma-like 1 (p107) | |
| 16673154 | 7.92E-04 | 1.4672061 | *NUF2* |  |  |
| 16844312 | 2.98E-04 | 1.46713 | *TOP2A* | topoisomerase (DNA) II alpha 170kDa | |
| 17043843 | 1.69E-05 | 1.4661124 | *TSPAN13* | tetraspanin 13 |  |
| 17010552 | 3.29E-04 | 1.4645225 | *TTK* |  |  |
| 16794719 | 3.37E-04 | 1.4644675 | *LTBP2* | latent transforming growth factor beta binding protein 2 | |
| 16964000 | 8.00E-06 | 1.4634355 | *TACC3* | transforming, acidic coiled-coil containing protein 3 | |
| 16857258 | 1.13E-04 | 1.4629185 | *UHRF1* | ubiquitin-like with PHD and ring finger domains 1 | |
| 16692636 | 3.53E-04 | 1.4608091 | *HIST2H2AB* | histone cluster 2, H2ab |  |
| 16922584 | 1.03E-05 | 1.4595555 | *CHAF1B* | chromatin assembly factor 1, subunit B (p60) | |
| 16841525 | 1.21E-06 | 1.4593428 | *HS3ST3A1* | heparan sulfate (glucosamine) 3-O-sulfotransferase 3A1 | |
| 16949264 | 0.001133 | 1.4584752 | *LOC344887* | NmrA-like family domain containing 1 pseudogene | |
| 16684898 | 0.007113 | 1.458375 |  |  |  |
| 17080749 | 5.56E-05 | 1.4578465 | *ATAD2* | ATPase family, AAA domain containing 2 | |
| 17102559 | 1.61E-04 | 1.4571413 |  |  |  |
| 16889251 | 1.28E-04 | 1.456395 | *SGOL2* | shugoshin-like 2 (S. pombe) |  |
| 16817017 | 4.58E-06 | 1.4557899 | *PLK1* | polo-like kinase 1 |  |
| 16705283 | 0.011521 | 1.4548297 |  |  |  |
| 16696533 | 0.001781 | 1.4540732 | *SNORD75* | small nucleolar RNA, C/D box 75 | |
| 16713309 | 3.63E-05 | 1.453823 | *FZD8* | frizzled homolog 8 (Drosophila) | |
| 16802519 | 1.82E-05 | 1.4531374 | *KIF23* | kinesin family member 23 |  |
| 16982635 | 8.95E-07 | 1.4523965 | *TRIP13* | thyroid hormone receptor interactor 13 | |
| 17005396 | 4.29E-04 | 1.451008 | *GMNN* | geminin, DNA replication inhibitor | |
| 16860159 | 0.001944 | 1.4503468 | *ZNF714* | zinc finger protein 714 |  |
| 16829369 | 5.40E-06 | 1.4502575 | *FANCA* | Fanconi anemia, complementation group A | |
| 16777278 | 3.40E-06 | 1.449391 | *SKA3* | spindle and kinetochore associated complex subunit 3 | |
| 16908728 | 1.43E-06 | 1.448883 | *PTPRN* | protein tyrosine phosphatase, receptor type, N | |
| 16666357 | 3.85E-06 | 1.4486343 | *AK5* | adenylate kinase 5 |  |
| 16810976 | 0.023006 | 1.4482999 | *SNORD18A* | small nucleolar RNA, C/D box 18A | |
| 17020019 | 8.24E-05 | 1.4481257 | *MCM3* | minichromosome maintenance complex component 3 | |
| 17051189 | 0.030258 | 1.447444 |  |  |  |
| 16913681 | 1.97E-05 | 1.4468647 | *FAM83D* | family with sequence similarity 83, member D | |
| 16904780 | 6.45E-08 | 1.4449311 | *SPC25* |  |  |
| 16828327 | 1.00E-06 | 1.444892 | *RFWD3* | ring finger and WD repeat domain 3 | |
| 16887840 | 3.97E-04 | 1.4448197 | *CDCA7* | cell division cycle associated 7 | |
| 16836492 | 1.39E-07 | 1.4438509 | *PRR11* | proline rich 11 |  |
| 17019805 | 5.08E-05 | 1.4437572 | *TNFRSF21* | tumor necrosis factor receptor superfamily, member 21 | |
| 17079220 | 4.50E-04 | 1.4432474 | *RAD54B* | RAD54 homolog B (S. cerevisiae) | |
| 17077826 | 6.89E-06 | 1.441346 | *MYBL1\|LOC645895* | v-myb myeloblastosis viral oncogene homolog (avian)-like 1 \| hypothetical LOC645895 | |
| 16905108 | 7.15E-05 | 1.4412539 | *DLX2* | distal-less homeobox 2 |  |
| 17060412 | 1.50E-04 | 1.4407904 | *MCM7* | minichromosome maintenance complex component 7 | |
| 16994618 | 3.07E-06 | 1.4406621 | *MYO10* | myosin X |  |
| 16722078 | 0.004213 | 1.4400047 |  |  |  |
| 16686796 | 4.47E-05 | 1.439833 | *STIL* | SCL/TAL1 interrupting locus |  |
| 16869588 | 3.48E-05 | 1.4395361 | *ASF1B* | ASF1 anti-silencing function 1 homolog B (S. cerevisiae) | |
| 17033478 | 0.003186 | 1.4381918 | *MSH5* | MutS protein homolog 5 |  |
| 16793225 | 2.77E-05 | 1.4366262 | *DLGAP5* | discs, large (Drosophila) homolog-associated protein 5 | |
| 16858714 | 1.04E-04 | 1.4364076 | *RNASEH2A* | ribonuclease H2, subunit A |  |
| 17105401 | 1.54E-04 | 1.4343415 | *CENPI* | centromere protein I |  |
| 16798812 | 0.030308 | 1.4334849 |  |  |  |
| 16775324 | 8.54E-05 | 1.4324551 | *C13orf34* | chromosome 13 open reading frame 34 | |
| 16912192 | 9.34E-05 | 1.4323994 | *GINS1* | GINS complex subunit 1 (Psf1 homolog) | |
| 17016360 | 0.006348 | 1.4323258 | *HIST1H4B* | histone cluster 1, H4b |  |
| 16810972 | 0.001712 | 1.4314364 | *SNORD18B* | small nucleolar RNA, C/D box 18B | |
| 16981502 | 0.002697 | 1.4312048 |  |  |  |
| 16932483 | 9.44E-04 | 1.4299954 |  |  |  |
| 16870821 | 7.66E-04 | 1.4294866 | *ZNF100* | zinc finger protein 100 |  |
| 17007459 | 4.92E-07 | 1.4274422 | *KIFC1* | kinesin family member C1 |  |
| 17041352 | 5.34E-07 | 1.4258809 |  |  |  |
| 16717412 | 3.18E-06 | 1.4254841 | *DNMBP* | dynamin binding protein |  |
| 17022150 | 4.84E-05 | 1.4253935 | *POPDC3* | popeye domain containing 3 | |
| 16736682 | 0.005879 | 1.42534 |  |  |  |
| 16663958 | 7.46E-06 | 1.4249259 | *KIF2C* | kinesin family member 2C |  |
| 16937505 | 4.43E-04 | 1.424456 | *FANCD2* | Fanconi anemia, complementation group D2 | |
| 17064679 | 8.88E-05 | 1.4237347 | *XRCC2* | X-ray repair complementing defective repair in Chinese hamster cells 2 | |
| 16953279 | 7.43E-05 | 1.4237239 | *CDC25A* | cell division cycle 25 homolog A (S. pombe) | |
| 16738657 | 0.006115 | 1.4237224 | *GLYATL2* | glycine-N-acyltransferase-like 2 | |
| 17088442 | 0.006313 | 1.423067 |  |  |  |
| 17005589 | 2.44E-04 | 1.4224958 | *HIST1H2AE* | histone cluster 1, H2ae |  |
| 17005603 | 0.002035 | 1.4222078 | *HIST1H2BI* | histone cluster 1, H2bi |  |
| 16803562 | 2.78E-04 | 1.4213139 | *CHRNA5* | cholinergic receptor, nicotinic, alpha 5 | |
| 16840902 | 3.75E-04 | 1.4196143 | *AURKB* | aurora kinase B |  |
| 16751709 | 4.78E-04 | 1.4192892 | *ESPL1* | extra spindle pole bodies homolog 1 (S. cerevisiae) | |
| 17009482 | 4.30E-06 | 1.4189482 | *CENPQ* | centromere protein Q |  |
| 17087716 | 0.001055 | 1.4182167 | *SMC2* | structural maintenance of chromosomes 2 | |
| 16807605 | 3.30E-04 | 1.4173254 | *OIP5* | Opa interacting protein 5 |  |
| 16787364 | 1.29E-05 | 1.4171402 | *TDP1* | tyrosyl-DNA phosphodiesterase 1 | |
| 16714998 | 3.15E-04 | 1.416917 | *DNA2* | DNA replication helicase 2 homolog (yeast) | |
| 16766318 | 1.76E-04 | 1.4168152 | *PRIM1* | primase, DNA, polypeptide 1 (49kDa) | |
| 16695741 | 1.74E-04 | 1.416301 | *OLFML2B* | olfactomedin-like 2B |  |
| 17068385 | 1.50E-04 | 1.4158719 | *GINS4* | GINS complex subunit 4 (Sld5 homolog) | |
| 16723422 | 4.38E-06 | 1.4156868 | *C11orf41* | chromosome 11 open reading frame 41 | |
| 17111219 | 8.28E-04 | 1.415143 | *SMC1A* | structural maintenance of chromosomes 1A | |
| 16918445 | 1.96E-05 | 1.4141349 | *E2F1* | E2F transcription factor 1 |  |
| 17112769 | 4.90E-05 | 1.4134663 | *GLA* | galactosidase, alpha |  |
| 16801557 | 1.05E-06 | 1.4132426 | *CCNB2* | cyclin B2 |  |
| 16912379 | 2.09E-06 | 1.4126234 | *TPX2* |  |  |
| 16767751 | 3.12E-06 | 1.411796 | *PHLDA1* | pleckstrin homology-like domain, family A, member 1 | |
| 17016499 | 0.002918 | 1.4115995 | *HIST1H1B* | histone cluster 1, H1b |  |
| 16802413 | 6.14E-05 | 1.411023 | *CORO2B* | coronin, actin binding protein, 2B | |
| 16870443 | 3.65E-05 | 1.4101552 | *CRLF1* | cytokine receptor-like factor 1 | |
| 17012379 | 8.85E-05 | 1.4098967 | *CENPW* | centromere protein W |  |
| 17104484 | 9.03E-04 | 1.4086558 | *KIF4A* | kinesin family member 4A |  |
| 16901226 | 0.002885 | 1.4086063 | *RFX8* | regulatory factor X, 8 |  |
| 16668960 | 6.76E-05 | 1.4080012 | *DCLRE1B* | DNA cross-link repair 1B |  |
| 16877762 | 7.86E-06 | 1.4046189 | *CENPO* | centromere protein O |  |
| 16870581 | 2.20E-04 | 1.4045484 | *NR2C2AP* | nuclear receptor 2C2-associated protein | |
| 16857192 | 5.05E-05 | 1.4034753 | *CHAF1A* | chromatin assembly factor 1, subunit A (p150) | |
| 17016363 | 5.41E-05 | 1.4025236 | *HIST1H3B* | histone cluster 1, H3b |  |
| 17005858 | 1.06E-04 | 1.4013972 | *HIST1H2AI* | histone cluster 1, H2ai |  |
| 16877956 | 7.33E-05 | 1.4013463 | *CENPA* | centromere protein A |  |
| 16863209 | 0.015551 | 1.3990327 |  |  |  |
| 16844354 | 0.003906 | 1.3987708 |  |  |  |
| 16966443 | 1.64E-05 | 1.3985633 |  |  |  |
| 17075776 | 2.60E-04 | 1.3980687 | *PBK* | PDZ binding kinase |  |
| 16668079 | 6.78E-05 | 1.3979508 | *GPSM2* | G-protein signaling modulator 2 | |
| 16928965 | 0.028565 | 1.3975471 |  |  |  |
| 16758336 | 1.07E-04 | 1.3973591 | *KNTC1* | kinetochore associated 1 |  |
| 16799517 | 5.22E-06 | 1.3968647 | *C15orf23* | chromosome 15 open reading frame 23 | |
| 16818600 | 0.001065 | 1.3962976 | *ORC6\|ORC6L* | origin recognition complex, subunit 6 \| origin recognition complex, subunit 6 like (yeast) | |
| 16804902 | 4.41E-04 | 1.3962367 | *BLM* | Bloom syndrome, RecQ helicase-like | |
| 17000439 | 5.68E-04 | 1.3954011 | *CDC25C* | cell division cycle 25 homolog C (S. pombe) | |
| 17117871 | 2.87E-04 | 1.3945111 | *HMGA1* | high mobility group AT-hook 1 | |
| 16781482 | 3.20E-06 | 1.3943948 | *PARP2* | poly (ADP-ribose) polymerase 2 | |
| 16768936 | 6.73E-04 | 1.3943437 | *LOC100128191* |  |  |
| 16918976 | 1.11E-05 | 1.3935627 | *DSN1* |  |  |
| 16706180 | 8.72E-05 | 1.3934205 | *PLAU* | antigen identified by monoclonal antibody Ki-67 | |
| 16971573 | 0.001805 | 1.3924582 | *MND1* | meiotic nuclear divisions 1 homolog (S. cerevisiae) | |
| 16929573 | 1.57E-04 | 1.3923547 | *MCM5* | minichromosome maintenance complex component 5 | |
| 16709936 | 2.01E-06 | 1.3918806 | *PPAPDC1A* | phosphatidic acid phosphatase type 2 domain containing 1A | |
| 16813206 | 9.54E-05 | 1.3918657 | *ANPEP* | alanyl (membrane) aminopeptidase | |
| 16987610 | 1.72E-04 | 1.3903106 | *RGMB* | RGM domain family, member B | |
| 16845172 | 5.55E-04 | 1.3901771 | *PSMC3IP* | PSMC3 interacting protein |  |
| 16747014 | 2.43E-04 | 1.3901045 | *RAD51AP1* | RAD51 associated protein 1 |  |
| 17007738 | 6.04E-06 | 1.3897429 | *SCUBE3* | signal peptide, CUB domain, EGF-like 3 | |
| 16828886 | 0.001402 | 1.3896917 | *GINS2* | GINS complex subunit 2 (Psf2 homolog) | |
| 17111998 | 5.07E-04 | 1.3896898 | *ERCC6L* | excision repair cross-complementing rodent repair deficiency, complementation group 6-like | |
| 16773946 | 3.23E-05 | 1.3889292 | *RFC3* | replication factor C (activator 1) 3, 38kDa | |
| 16673126 | 1.72E-04 | 1.3883387 | *RGS4* | regulator of G-protein signaling 4 | |
| 16988703 | 1.08E-04 | 1.3880506 | *LMNB1* | lamin B1 |  |
| 17045198 | 7.06E-06 | 1.3875617 | *ANLN* | anillin, actin binding protein | |
| 16817647 | 6.50E-06 | 1.3867064 | *KIF22* | kinesin family member 22 |  |
| 16909277 | 5.82E-05 | 1.3858197 | *SPHKAP* | SPHK1 interactor, AKAP domain containing | |
| 16698023 | 1.38E-05 | 1.3856374 | *UBE2T* | ubiquitin-conjugating enzyme E2T (putative) | |
| 17084904 | 1.88E-04 | 1.3839734 | *MELK* | maternal embryonic leucine zipper kinase | |
| 16947556 | 3.89E-06 | 1.3839481 | *SMC4* | structural maintenance of chromosomes 4 | |
| 17117613 | 0.01033 | 1.3837898 | *ANKRD10* | ankyrin repeat domain 10 |  |
| 16766137 | 6.88E-04 | 1.3810834 | *TIMELESS* | timeless homolog (Drosophila) | |
| 16802204 | 7.16E-05 | 1.3809249 | *ZWILCH* | Zwilch, kinetochore associated, homolog (Drosophila) | |
| 16845410 | 2.09E-04 | 1.3805144 | *ETV4* | ets variant 4 |  |
| 17000518 | 0.007735 | 1.3791267 | *HSPA9* | heat shock 70kDa protein 9 (mortalin) | |
| 17005532 | 1.27E-04 | 1.3790619 | *HIST1H3A* | histone cluster 1, H3a |  |
| 17024980 | 3.88E-05 | 1.3787625 | *FBXO5* | F-box protein 5 |  |
| 16749875 | 0.011769 | 1.3776003 | *ALG10* | asparagine-linked glycosylation 10, alpha-1,2-glucosyltransferase homolog (S. pombe) | |
| 16705159 | 8.90E-05 | 1.3772322 | *CDK1* | cyclin-dependent kinase 1 |  |
| 16835158 | 5.83E-04 | 1.3771718 | *ITGB3* | integrin, beta 3 (platelet glycoprotein IIIa, antigen CD61) | |
| 16783836 | 6.04E-04 | 1.3770986 | *FANCM* | Fanconi anemia, complementation group M | |
| 17005578 | 0.002485 | 1.3766875 | *HIST1H2BE* | histone cluster 1, H2be |  |
| 17105332 | 0.00706 | 1.3764505 | *SRPX2* | sushi-repeat-containing protein, X-linked 2 | |
| 17059828 | 0.006892 | 1.3763523 | *TFPI2* | tissue factor pathway inhibitor 2 | |
| 17109464 | 7.99E-05 | 1.3760478 | *SCML2* | sex comb on midleg-like 2 (Drosophila) | |
| 16811638 | 2.49E-04 | 1.3755302 | *SEMA7A* | semaphorin 7A, GPI membrane anchor (John Milton Hagen blood group) | |
| 16714504 | 5.52E-04 | 1.3750478 | *ZWINT* | ZW10 interactor |  |
| 16735545 | 2.59E-04 | 1.3748852 | *NRIP3* | nuclear receptor interacting protein 3 | |
| 16722720 | 2.78E-04 | 1.3748462 | *NAV2* | neuron navigator 2 |  |
| 17005600 | 0.001943 | 1.3738668 | *HIST1H2BH* | histone cluster 1, H2bh |  |
| 16985599 | 8.98E-05 | 1.3729279 | *CCNB1* | cyclin B1 |  |
| 16692583 | 4.07E-05 | 1.3724012 | *FAM72C* | family with sequence similarity 72, member C | |
| 16726790 | 3.38E-05 | 1.3723764 | *POLA2* | polymerase (DNA directed), alpha 2 (70kD subunit) | |
| 17085685 | 2.18E-05 | 1.3718759 | *TJP2* | tight junction protein 2 (zona occludens 2) | |
| 16970762 | 3.39E-06 | 1.3716288 | *PCDH10* | protocadherin 10 |  |
| 16780793 | 0.005034 | 1.3714961 | *C13orf27* | chromosome 13 open reading frame 27 | |
| 17021845 | 0.001642 | 1.3707877 | *MMS22L\|C6orf167* | MMS22-like, DNA repair protein \| chromosome 6 open reading frame 167 | |
| 16663514 | 1.79E-05 | 1.3706098 | *CDC20* | cell division cycle 20 homolog (S. cerevisiae) | |
| 16960844 | 2.01E-04 | 1.3701333 | *VEPH1* | ventricular zone expressed PH domain homolog 1 (zebrafish) | |
| 16821239 | 6.68E-06 | 1.3692095 | *CENPN* | centromere protein N |  |
| 16855820 | 1.80E-04 | 1.3684827 | *RTTN* | rotatin |  |
| 16964050 | 4.42E-05 | 1.3684516 | *WHSC1* | Wolf-Hirschhorn syndrome candidate 1 | |
| 16886491 | 8.02E-04 | 1.3680308 | *TNFAIP6* | tumor necrosis factor, alpha-induced protein 6 | |
| 16908171 | 3.48E-04 | 1.3665456 | *MARCH4-* | membrane-associated ring finger (C3HC4) 4 | |
| 16834812 | 2.97E-04 | 1.3664454 | *DBF4B* | DBF4 homolog B (S. cerevisiae) | |
| 16866951 | 0.002317 | 1.3661671 | *LMNB2* | lamin B2 |  |
| 16860418 | 1.71E-04 | 1.3656596 | *CCNE1* | cyclin E1 |  |
| 16868576 | 1.72E-05 | 1.3641356 | *DNMT1* | DNA (cytosine-5-)-methyltransferase 1 | |
| 16687188 | 2.77E-04 | 1.363908 | *ORC1\|ORC1L* | origin recognition complex, subunit 1 \| origin recognition complex, subunit 1-like (S. cerevisiae) | |
| 17112060 | 6.28E-05 | 1.36375 | *PHKA1* | phosphorylase kinase, alpha 1 (muscle) | |
| 17010246 | 0.001493 | 1.3635504 | *KCNQ5* | potassium voltage-gated channel, KQT-like subfamily, member 5 | |
| 16854792 | 9.69E-04 | 1.3631932 | *LOC647946* |  |  |
| 16750761 | 1.93E-04 | 1.362042 | *TROAP* | trophinin associated protein (tastin) | |
| 16745236 | 2.88E-05 | 1.3619493 | *H2AFX* | H2A histone family, member X | |
| 17050591 | 1.22E-04 | 1.3617994 | *MET* | met proto-oncogene (hepatocyte growth factor receptor) | |
| 16861318 | 2.10E-04 | 1.3617278 | *WDR62* | WD repeat domain 62 |  |
| 16688799 | 0.001107 | 1.3607749 | *ELTD1* | EGF, latrophilin and seven transmembrane domain containing 1 | |
| 16688386 | 4.94E-04 | 1.360442 | *DEPDC1* | DEP domain containing 1 |  |
| 16683712 | 3.77E-05 | 1.3602648 | *C1orf135* | chromosome 1 open reading frame 135 | |
| 16808793 | 0.00311 | 1.358902 | *FBN1* | fibrillin 1 |  |
| 16929562 | 5.94E-05 | 1.3584591 | *HMOX1* | heme oxygenase (decycling) 1 | |
| 17110367 | 0.017562 | 1.3580163 | *MIR222* | microRNA 222 |  |
| 16737783 | 0.001277 | 1.357754 | *CKAP5* | cytoskeleton associated protein 5 | |
| 16997676 | 0.002309 | 1.3577034 | *MTRNR2L2* | MT-RNR2-like 2 |  |
| 16799724 | 9.03E-05 | 1.3567566 | *DLL4* | delta-like 4 (Drosophila) |  |
| 16775014 | 5.42E-05 | 1.3566798 | *CKAP2* | cytoskeleton associated protein 2 | |
| 16920548 | 4.26E-06 | 1.3564137 | *AURKA* | aurora kinase A |  |
| 16795304 | 6.39E-05 | 1.3553759 | *C14orf145* | chromosome 14 open reading frame 145 | |
| 17096205 | 1.40E-04 | 1.3538959 | *ZNF367* | zinc finger protein 367 |  |
| 16725806 | 6.57E-04 | 1.3536028 | *INCENP* | inner centromere protein antigens 135/155kDa | |
| 16883107 | 0.021698 | 1.3535811 | *ANKRD36* | ankyrin repeat domain 36 |  |
| 16667206 | 0.001098 | 1.3535526 | *CCDC18* | coiled-coil domain containing 18 | |
| 16838359 | 1.09E-05 | 1.3529451 | *BIRC5* | baculoviral IAP repeat-containing 5 | |
| 16714747 | 4.71E-04 | 1.3524886 | *RTKN2* | rhotekin 2 |  |
| 17067314 | 2.44E-05 | 1.3521199 | *SCARA3* | scavenger receptor class A, member 3 | |
| 17051965 | 1.81E-04 | 1.3515058 | *NUP205* | nucleoporin 205kDa |  |
| 16956714 | 3.10E-05 | 1.350835 | *DCBLD2* | discoidin, CUB and LCCL domain containing 2 | |
| 16870131 | 3.30E-05 | 1.3499194 | *HAUS8* | HAUS augmin-like complex, subunit 8 | |
| 16861604 | 1.88E-05 | 1.34959 | *SIPA1L3* | signal-induced proliferation-associated 1 like 3 | |
| 16989636 | 4.30E-05 | 1.3492081 | *KIF20A* | kinesin family member 20A |  |
| 16755692 | 3.71E-05 | 1.3474903 | *GAS2L3* | growth arrest-specific 2 like 3 | |
| 16834056 | 8.08E-04 | 1.3462274 | *CDC6* | cell division cycle 6 homolog (S. cerevisiae) | |
| 16779546 | 2.89E-04 | 1.3460568 | *DIAPH3* | diaphanous homolog 3 (Drosophila) | |
| 16799426 | 1.23E-05 | 1.3460051 | *BUB1B\|PAK6* | budding uninhibited by benzimidazoles 1 homolog beta (yeast) \| p21 protein (Cdc42/Rac)-activated kinase 6 | |
| 16845736 | 1.47E-04 | 1.3459818 | *GJC1* | gap junction protein, gamma 1, 45kDa | |
| 17078190 | 3.21E-04 | 1.3455744 | *TRPA1* | transient receptor potential cation channel, subfamily A, member 1 | |
| 16676983 | 0.00597 | 1.3451931 | *G0S2* | G0/G1switch 2 |  |
| 16746379 | 3.75E-05 | 1.344894 | *NCAPD3* | non-SMC condensin II complex, subunit D3 | |
| 16968331 | 1.30E-04 | 1.3422781 | *FGF5* | fibroblast growth factor 5 |  |
| 16760621 | 3.44E-05 | 1.3420709 | *CDCA3* | cell division cycle associated 3 | |
| 17016512 | 3.47E-04 | 1.3412573 | *HIST1H2AM* | histone cluster 1, H2am |  |
| 16804539 | 9.23E-06 | 1.3410201 | *ABHD2* | abhydrolase domain containing 2 | |
| 16673557 | 4.00E-04 | 1.3409282 | *C1orf112* | chromosome 1 open reading frame 112 | |
| 16934568 | 7.54E-05 | 1.3406837 | *FOXRED2* | FAD-dependent oxidoreductase domain containing 2 | |
| 17063221 | 0.002521 | 1.3406413 | *FAM180A* | family with sequence similarity 180, member A | |
| 16970409 | 3.52E-04 | 1.3404351 | *SPATA5* | spermatogenesis associated 5 | |
| 16803008 | 1.00E-03 | 1.340134 | *LOC440288* | similar to FLJ16518 protein |  |
| 17079588 | 0.001965 | 1.3401216 | *FBXO43* | F-box protein 43 |  |
| 16945993 | 0.001023 | 1.3392249 | *PPP2R3A* | protein phosphatase 2, regulatory subunit B'', alpha | |
| 16703478 | 2.77E-06 | 1.3384404 | *MASTL* | microtubule associated serine/threonine kinase-like | |
| 16924966 | 3.10E-05 | 1.3382341 | *C21orf45* | chromosome 21 open reading frame 45 | |
| 17089898 | 5.02E-05 | 1.3378499 | *NUP188* | nucleoporin 188kDa |  |
| 16968797 | 1.22E-04 | 1.3371733 | *HERC3* | hect domain and RLD 3 |  |
| 16701317 | 0.049123 | 1.336822 | *NCRNA00201* | non-protein coding RNA 201 |  |
| 17024002 | 4.61E-05 | 1.336259 | *FAM54A* | family with sequence similarity 54, member A | |
| 16860175 | 1.41E-04 | 1.3361607 | *ZNF738* | zinc finger protein 738 |  |
| 16772625 | 1.81E-05 | 1.3357888 | *POLE* | polymerase (DNA directed), epsilon | |
| 17093397 | 0.001404 | 1.3357342 | *KIF24* | kinesin family member 24 |  |
| 16979515 | 9.17E-07 | 1.3356394 | *CCNA2* | cyclin A2 |  |
| 16658289 | 0.001028 | 1.3354257 | *AJAP1* | adherens junctions associated protein 1 | |
| 17016366 | 0.031862 | 1.3350734 | *HIST1H2AB* | histone cluster 1, H2ab |  |
| 16935517 | 6.06E-05 | 1.3346744 | *CENPM* | centromere protein M |  |
| 16700034 | 8.24E-05 | 1.3338933 | *LIN9* | lin-9 homolog (C. elegans) |  |
| 16661342 | 3.40E-07 | 1.3333607 | *TRNP1* | TMF1-regulated nuclear protein 1 | |
| 16760048 | 1.17E-05 | 1.3326634 | *FOXM1* | forkhead box M1 |  |
| 17005582 | 9.82E-04 | 1.3322979 | *HIST1H2BF* | histone cluster 1, H2bf |  |
| 16808876 | 4.28E-04 | 1.3313754 | *CEP152* | centrosomal protein 152kDa |  |
| 16702685 | 8.26E-06 | 1.3310782 | *SUV39H2* | suppressor of variegation 3-9 homolog 2 (Drosophila) | |
| 16970231 | 2.17E-05 | 1.3306504 | *EXOSC9* | exosome component 9 |  |
| 16997199 | 4.55E-05 | 1.330552 | *ENC1* | ectodermal-neural cortex 1 (with BTB-like domain) | |
| 17117724 | 0.005614 | 1.3303595 | *PSMD3* | proteasome (prosome, macropain) 26S subunit, non-ATPase, 3 | |
| 16692477 | 0.039484 | 1.3298004 | *NBPF10* | neuroblastoma breakpoint family, member 10 | |
| 16998456 | 0.003508 | 1.3295946 | *FLJ35946* |  |  |
| 17000235 | 5.18E-04 | 1.3294946 | *SPOCK1* | sparc/osteonectin, cwcv and kazal-like domains proteoglycan (testican) 1 | |
| 16909401 | 3.69E-05 | 1.3293939 | *SLC16A14* | solute carrier family 16, member 14 (monocarboxylic acid transporter 14) | |
| 16707551 | 1.40E-04 | 1.3291577 | *CEP55* | centrosomal protein 55kDa |  |
| 16771242 | 4.99E-04 | 1.3286488 | *PXN* | paxillin |  |
| 16916958 | 9.48E-05 | 1.3281716 | *PCNA* | proliferating cell nuclear antigen | |
| 16870828 | 0.014454 | 1.3277987 | *ZNF43* | zinc finger protein 43 |  |
| 16806732 | 0.028408 | 1.3258257 | *LOC100288615* | WAS protein homolog associated with actin, golgi membranes and microtubules pseudogene | |
| 16821869 | 1.69E-04 | 1.3253098 | *CDT1* | chromatin licensing and DNA replication factor 1 | |
| 17102210 | 0.003486 | 1.325055 | *PDK3* | pyruvate dehydrogenase kinase, isozyme 3 | |
| 16676965 | 4.46E-04 | 1.324978 | *CAMK1G* | calcium/calmodulin-dependent protein kinase IG | |
| 17023658 | 0.003988 | 1.3241454 | *MOXD1* | monooxygenase, DBH-like 1 |  |
| 16991859 | 2.63E-04 | 1.3236966 | *HMMR* | hyaluronan-mediated motility receptor (RHAMM) | |
| 17112357 | 1.20E-04 | 1.3226234 | *ZCCHC5* | zinc finger, CCHC domain containing 5 | |
| 16676526 | 9.02E-04 | 1.3225644 | *FAM72A* | family with sequence similarity 72, member A | |
| 16899401 | 0.00114 | 1.3224849 | *TACR1* | tachykinin receptor 1 |  |
| 17005596 | 0.031044 | 1.3219564 | *HIST1H4F* | histone cluster 1, H4f |  |
| 16799793 | 4.21E-05 | 1.3219094 | *NUSAP1* | nucleolar and spindle associated protein 1 | |
| 16727363 | 0.002676 | 1.3218067 | *CNIH2* | cornichon homolog 2 (Drosophila) | |
| 17112191 | 4.31E-04 | 1.3211497 | *MIR545* | microRNA 545 |  |
| 16669212 | 2.74E-04 | 1.3193983 | *TTF2* | transcription termination factor, RNA polymerase II | |
| 17075314 | 2.58E-04 | 1.319078 | *REEP4* | receptor accessory protein 4 |  |
| 17047965 | 4.00E-04 | 1.3186255 | *DBF4* |  |  |
| 17076867 | 0.00657 | 1.318286 | *PRKDC* | protein kinase, DNA-activated, catalytic polypeptide | |
| 16924521 | 0.02076 | 1.3181512 | *C21orf71* | chromosome 21 open reading frame 71 | |
| 16740282 | 0.001215 | 1.3177292 | *CDCA5\|LOC256676* | cell division cycle associated 5 \| hypothetical protein | |
| 17057718 | 6.70E-04 | 1.3176736 | *FIGNL1* | fidgetin-like 1 |  |
| 16788883 | 0.005013 | 1.3174676 | *DYNC1H1* | dynein, cytoplasmic 1, heavy chain 1 | |
| 16927052 | 2.25E-04 | 1.317431 | *CDC45* | cell division cycle 45 homolog (S. cerevisiae) | |
| 16941691 | 0.004282 | 1.3172015 | *SNORD69* | small nucleolar RNA, C/D box 69 | |
| 16811858 | 0.00163 | 1.3170373 | *CSPG4* | chondroitin sulfate proteoglycan 4 | |
| 17064105 | 0.001072 | 1.316651 | *EZH2* | enhancer of zeste homolog 2 (Drosophila) | |
| 16661117 | 7.49E-06 | 1.3165967 | *CCDC21* | coiled-coil domain containing 21 | |
| 17080595 | 0.003051 | 1.3165729 | *DSCC1* | defective in sister chromatid cohesion 1 homolog (S. cerevisiae) | |
| 16979389 | 8.55E-05 | 1.3159227 | *MAD2L1* | MAD2 mitotic arrest deficient-like 1 (yeast) | |
| 16723353 | 0.001344 | 1.3158324 | *DEPDC7* | DEP domain containing 7 |  |
| 16959325 | 5.54E-05 | 1.3157474 | *TOPBP1* | topoisomerase (DNA) II binding protein 1 | |
| 16696387 | 8.22E-04 | 1.3155941 | *MIR214\|MIR199A2* | microRNA 214 \| microRNA 199a-2 | |
| 16852683 | 3.01E-04 | 1.3152694 | *PMAIP1* | phorbol-12-myristate-13-acetate-induced protein 1 | |
| 16925239 | 8.91E-05 | 1.3147303 | *DONSON\|ATP5O* | downstream neighbor of SON \| ATP synthase, H+ transporting, mitochondrial F1 complex, O subunit | |
| 16962911 | 0.002103 | 1.3145785 | *LRRC15* | leucine rich repeat containing 15 | |
| 16855180 | 7.81E-04 | 1.3141181 | *SNORD58C\|U58* | small nucleolar RNA, C/D box 58C \| U58 small nucleolar RNA | |
| 16990862 | 0.003395 | 1.3134664 | *ABLIM3* | actin binding LIM protein family, member 3 | |
| 17046470 | 0.014209 | 1.3115807 | *ZNF107* | zinc finger protein 107 |  |
| 16830173 | 2.06E-04 | 1.3110404 | *FAM64A* | family with sequence similarity 64, member A | |
| 16892521 | 0.038313 | 1.3100107 | *SCARNA5* | small Cajal body-specific RNA 5 | |
| 16784215 | 6.76E-05 | 1.3095349 | *GPR137C* | G protein-coupled receptor 137C | |
| 16922501 | 2.31E-05 | 1.3082378 | *DOPEY2* | dopey family member 2 |  |
| 16826230 | 1.09E-04 | 1.3078601 | *NETO2* | neuropilin (NRP) and tolloid (TLL)-like 2 | |
| 16777502 | 2.84E-04 | 1.3078192 | *CENPJ* | centromere protein J |  |
| 16948021 | 7.68E-04 | 1.3075238 | *ECT2* | epithelial cell transforming sequence 2 oncogene | |
| 17005586 | 3.24E-04 | 1.3072729 | *HIST1H4E* | histone cluster 1, H4e |  |
| 16834711 | 8.92E-05 | 1.3067538 | *C17orf53* | chromosome 17 open reading frame 53 | |
| 16972616 | 1.47E-04 | 1.3063117 | *NEIL3* | nei endonuclease VIII-like 3 (E. coli) | |
| 17093595 | 6.64E-05 | 1.3063079 | *FANCG* | Fanconi anemia, complementation group G | |
| 17063311 | 6.40E-05 | 1.3040013 | *CREB3L2* | cAMP responsive element binding protein 3-like 2 | |
| 16736638 | 0.001422 | 1.3031577 | *E2F8* | E2F transcription factor 8 |  |
| 16728261 | 5.73E-05 | 1.3028959 | *CCND1* | cyclin D1 |  |
| 16838049 | 7.72E-05 | 1.3026584 | *FAM100B* | family with sequence similarity 100, member B | |
| 16845349 | 4.48E-04 | 1.3014024 | *BRCA1\|LOC100505899* | breast cancer 1, early onset \| hypothetical LOC100505899 | |
| 17102230 | 8.32E-04 | 1.3003304 | *POLA1* | polymerase (DNA directed), alpha 1, catalytic subunit | |
| 16692724 | 8.62E-05 | 1.3002053 | *ANP32E* | acidic (leucine-rich) nuclear phosphoprotein 32 family, member E | |
